# CREB3L1-Associated Stromal Expression During Mechanical Remodeling of the Mouse Zygomaticomaxillary Suture

**DOI:** 10.3390/biology15211918

**Published:** 2026-10-23

**Authors:** Sirui Tang, Yujie Chen, Zhenmin Zhao

**Affiliations:** 1 Department of Plastic Surgery, Peking University People’s Hospital, No. 11 Xizhimen South Street, Xicheng District, Beijing 100033, China; sirui0299@outlook.com; 2 Department of Plastic Surgery, Peking University Third Hospital, No. 49 North Garden Road, Haidian District, Beijing 100191, China

**Keywords:** CREB3L1, bone physiology, zygomaticomaxillary suture, mechanical loading, stromal cells, extracellular matrix, single-cell RNA sequencing

## Abstract

CREB3L1 expression during mechanical suture remodeling remains incompletely characterized. We examined transcripts and staining in distracted zygomaticomaxillary sutures (ZMS) from 4-week-old male C57BL/6J mice and expanded ZMS-derived cultures. Archived microscopy, four single-cell libraries, qPCR and micro-CT measurements were evaluated separately. Each single-cell condition/day represented one biological sample. Author clarification enabled micro-CT reanalysis of 61 animals. Exact tests used separate Holm corrections for tissue qPCR, culture-vessel qPCR and micro-CT. Compared with sham controls (SCs), distraction (DO) was associated with higher micro-CT bone volume at day 14 (Holm-adjusted *p* = 0.0346), whereas regional bone mineral density was lower in DO at days 3 and 28 (Holm-adjusted *p* = 0.0227 and 0.0216, respectively). With four animals per group, tissue *Creb3l1* and *Spp1* mean estimates were lower than same-day sham references on day 1 and higher on day 3; none of the 11 tissue qPCR comparisons was significant. Two static dishes and three stretch chambers came from one pooled six-mouse preparation in one experiment. After three days of stretch, *Creb3l1* and *Spp1* mean differences were −0.09 and +0.11 log2 units; both adjusted *p* values were 1.000. These preliminary structural and expression observations do not establish CREB3L1 activation or causality. Small samples, unreplicated single-cell conditions and incomplete imaging/batch metadata require independent validation.

## Simple Summary

Craniofacial sutures connect neighboring skull bones and contain cells involved in growth and repair. We examined the expression of CREB3L1, a protein linked to matrix production, in mechanically distracted mouse sutures and in expanded suture-derived cells exposed to stretch. Tissue gene measurements and single-cell profiles provided preliminary expression observations. Average tissue *Creb3l1* and *Spp1* expression was lower than the same-day sham reference on day 1 and higher on day 3, but these comparisons were not statistically significant. Micro-CT showed higher bone volume in distracted sutures at day 14, whereas regional bone mineral density was lower at days 3 and 28. The small culture series also did not establish a reproducible response to stretch. Differences in loading, tissue composition, culture expansion and sampling time may contribute to the observed patterns. The study supplies expression-level observations to guide experiments testing CREB3L1 processing and matrix production; it does not establish protein activation or a causal role in bone formation.

## 1. Introduction

Craniofacial sutures are fibrous interfaces that connect adjacent bones while accommodating skull growth. Distinct stem-cell populations within these interfaces contribute to calvarial mineralization and maintenance of suture patency [1]. Mechanical loading can alter the behavior of these cells and the surrounding extracellular matrix, making sutures a useful setting in which to examine the relationship between physical demand and skeletal adaptation. An early transcriptional response, however, is distinct from the subsequent production, organization and mineralization of matrix.

Trans-sutural distraction osteogenesis applies tensile force across a suture to promote craniofacial remodeling. Studies of *Gli1*-positive suture cells demonstrate a cellular contribution to expansion-associated bone remodeling [2]. More recently, experiments in calvarial suture cells identified *Mkx* as a stretch-responsive regulator that restrains osteogenic differentiation [3]. These observations show that mechanical responses can involve both bone-forming and niche-preserving programs. How matrix-associated transcription accompanies the early response of the zygomaticomaxillary suture (ZMS) remains less well defined.

CREB3L1, also termed OASIS, is an endoplasmic-reticulum membrane-associated transcription factor. Recent experiments in odontoblast-lineage cells connect CREB3L1 with matrix-protein production, secretion and dentin deposition [4]. Single-cell analysis of human femoral-head osteoblasts from one donor placed the highest inferred CREB3L1 regulon activity in mature osteoblasts [5]. This suggests a cell-state context for examining CREB3L1-associated transcripts, without establishing biochemical activation in loaded sutures. It is therefore a candidate for investigating the demands placed on matrix-producing cells during suture loading. Expression and activation must nevertheless be distinguished: *Creb3l1* mRNA and total CREB3L1 immunoreactivity do not reveal whether the protein has undergone proteolytic processing and nuclear translocation. Establishing its expression distribution in the suture is an initial step toward testing these mechanisms.

The loaded suture also contains immune and vascular cells that may modify stromal responses. Neutrophil-derived oncostatin M has been implicated in force-induced craniofacial bone regeneration [6]. Such paracrine inputs could accompany a CREB3L1-associated response without directly regulating it. Comparing observations in whole tissue with those in expanded suture-derived cultures can help identify questions about this context, although differences in cell composition and mechanical exposure prevent the two systems from being treated as equivalent models.

We examined CREB3L1-associated transcript and staining patterns in mechanically distracted mouse ZMS tissue and in expanded suture-derived stromal cultures exposed to cyclic stretch. Tissue qPCR, existing single-cell profiles and microscopy were assessed as distinct evidence components. *Creb3l1* and *Spp1* provided shared transcript endpoints for tissue and culture observations. The objective was to describe preliminary expression patterns and identify focused questions for subsequent activation and functional studies, rather than to establish a CREB3L1-mediated remodeling mechanism.

## 2. Materials and Methods

### 2.1. Study Design and Data Provenance

**Scheme 1:**
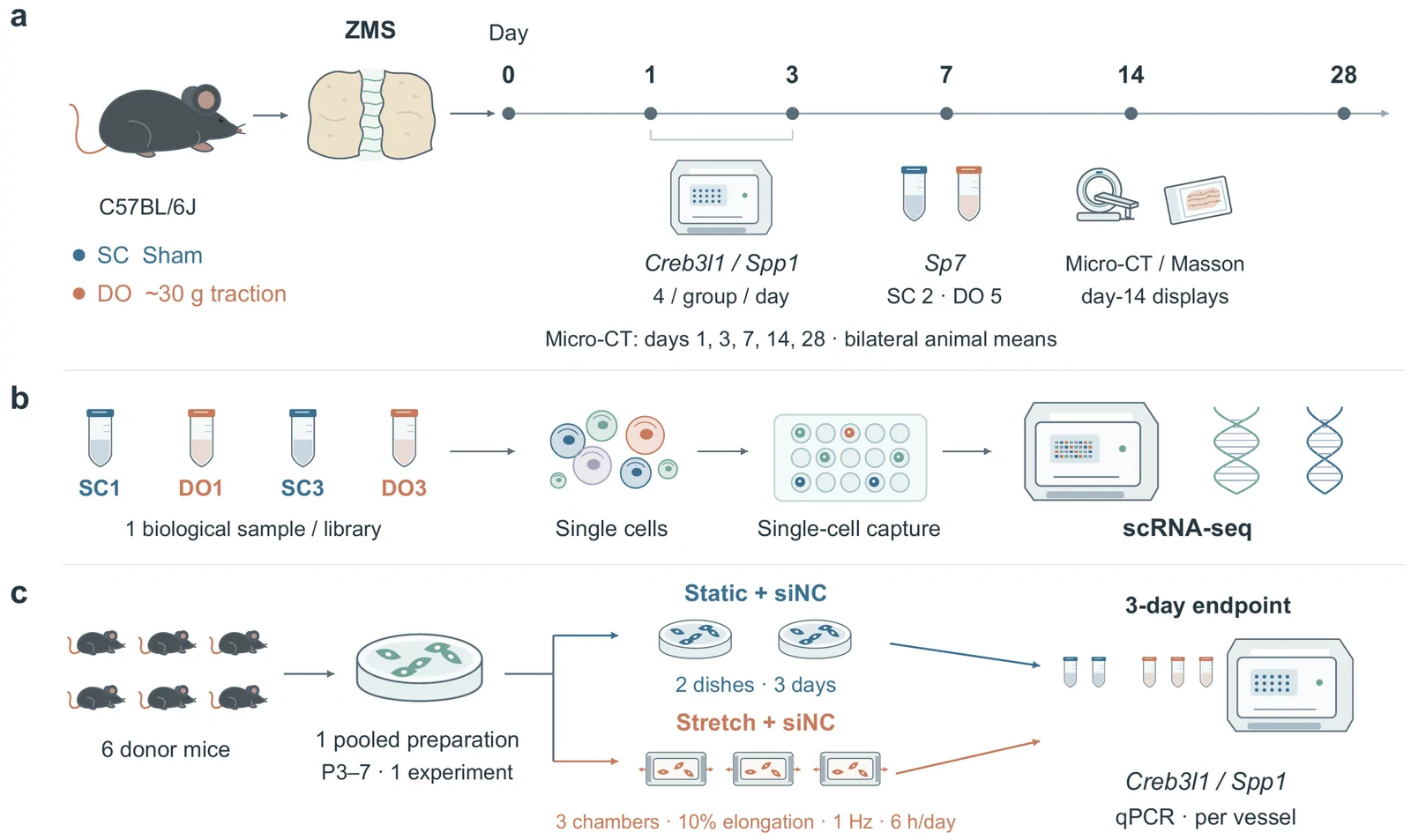
Experimental design and units of observation. (**a**) Tissue sampling in 4-week-old male C57BL/6J mice after sham surgery or nominal 30-g ZMS distraction. Tissue *Creb3l1*/*Spp1* qPCR concerns days 1 and 3 (4 animals/group), *Sp7* day 7 (2 SC/5 DO), and *Osm*/*Lifr*/*Osmr* days 1 and 3 with endpoint-specific counts in Table 1. Displayed micro-CT/Masson morphology concerns day 14; quantitative micro-CT measurements span days 1, 3, 7, 14 and 28, with animal counts in Table S5. Supplementary morphology spans days 1–28. (**b**) Four single-cell libraries: SC1, DO1, SC3 and DO3, each representing one biological sample and overlapping the dataset described in related publications. (**c**) For the reported qPCR experiment, tissue from six donor mice was pooled to establish one cell preparation, expanded through passages 3–7. Two separate static dishes (SC1/SC2) and three separately treated stretch chambers (3D-1/2/3), all receiving negative-control siRNA, were studied in one experiment. Each vessel supplied one RNA sample and two qPCR wells per target; this is vessel-level replication within one pooled preparation. Stretch was 10% elongation, 1 Hz and 6 h/day for 3 days. Targeting-siRNA images are qualitative supplementary records. Endpoint counts must not be summed as independent cohorts. The diagram depicts performed components, not proposed activation or rescue experiments. Blue and orange identify the control and mechanical-loading conditions, respectively; the numerals in SC1/DO1 and SC3/DO3 indicate sampling days 1 and 3.

This exploratory study examined sham and mechanically distracted ZMS tissue and expanded suture-derived stromal cultures. Scheme 1 summarizes the experimental components and their units of observation, and Table 1 links each component to its readouts. Tissue *Spp1*/*Creb3l1* qPCR and single-cell profiles concern days 1 and 3; *Sp7* qPCR concerns day 7; and the principal micro-CT and Masson comparisons concern day 14. Supplementary morphology spans days 1, 3, 7, 14 and 28. Tissue samples, sequencing libraries and culture vessels were evaluated separately. Day-specific tissue and micro-CT groups used different animals rather than longitudinal measurements, and the six donors contributing to the pooled qPCR culture preparation do not constitute six independent culture preparations.

The experimental work was conducted within the research program documented in Chen’s doctoral dissertation [7] and the related studies by Sun et al. and Lin et al. [6,8]. The present report addresses CREB3L1-associated expression within that program. Its single-cell material overlaps the dataset examined in the two publications; the figures retain selected existing visualizations of that material. Available source panels were compared with those publications during figure selection, and directly duplicated displays were not presented as new results. The tissue and culture observations are complementary components of the same program, rather than an independent cohort or replication of those studies. Experimental procedures needed to interpret this report are described below.

### 2.2. Animals, Distraction and Tissue Assessment

Four-week-old male C57BL/6J mice weighing 13–15 g were used. The dissertation records an initial 46-animal allocation (23 per group). During revision, the author clarified that this covered part of the study and that the archived micro-CT workbook also includes animals studied subsequently under the same conditions. The eligible micro-CT records represent 61 animals (31 SC, 30 DO); individual batch assignments and overlap with the other endpoints were not recovered. These counts are therefore reported by endpoint and are not summed into a total cohort size. Under pentobarbital sodium anesthesia at 80 mg/kg, a nickel-titanium W-shaped spring was positioned between the temporal and nasal-maxillary regions. Compression from 12 to 7 mm generated a nominal traction force of approximately 30 g. Sham animals underwent exposure and drilling without spring placement. Random allocation was described in the experimental record, but sequence generation, endpoint allocation and blinding were not documented. Mice were housed in the specific-pathogen-free facility of Peking University Health Science Center and received standard chow.

ZMS tissue and adjacent bone were collected after euthanasia by pentobarbital overdose. Sham and distraction samples were collected at the same designated time points: days 1, 3, 7, 14 and 28. The main anatomical comparison uses day-14 micro-CT reconstructions and Masson sections, with additional H&E histology in Figure S1. The experimental record specifies Quantum FX micro-CT imaging (PerkinElmer, Waltham, MA, USA) at 90 kV, 100 μA and 10-μm voxel resolution, with reconstruction in Mimics 20.0.0.691 (Materialise, Leuven, Belgium). Recovered scan headers specify 160 μA, but could not be linked unambiguously to the displayed reconstructions or quantitative workbook. Acquisition settings for the retained displays therefore remain unverified.

During revision, the author clarified the archived micro-CT metadata. MEAN denotes mean CT intensity in HU over a fixed-size box/cuboid centered on the ZMS, including bone and non-bone voxels. VOL denotes thresholded bone volume (BV, mm^3^). Regional bone mineral density (BMD, mg HA/cm^3^) was calculated as (mean HU + 739.11)/1.3487 using a standard-phantom calibration recalled by the author. Both sides were analyzed using the same region-of-interest (ROI) rule and one fixed bone-segmentation threshold. Numeric ROI dimensions, the exact HU threshold, the original phantom measurements and specimen-linked scanner settings were not recovered. The archived measurements can therefore be reanalyzed, but segmentation and calibration cannot be independently reproduced from the available records.

The ordinary ZMS-SC/DO workbook subset contains 66 labeled rows: four have no numerical measurements, and 62 contain both endpoints. Author-confirmed sample identities yield 61 different animals, with independent animals at each time point. All single entries are already bilateral animal-level means except ZMS-DO1-3, whose two rows represent the left and right sides; these were averaged to provide one animal-level value. SC/DO animal counts are 6/6, 7/5, 6/7, 6/6 and 6/6 at days 1, 3, 7, 14 and 28, respectively. GANT and CS records concern other experimental components and were not included; missing values were not imputed. These units, side-aggregation rules and measurement definitions were clarified retrospectively by the author, rather than inferred from numeric suffixes or recovered acquisition metadata.

Paraffin sections were processed for hematoxylin and eosin or Masson staining. The displayed fields describe anatomy and matrix staining and were not counted as independent animal-level measurements.

For H&E, paraffin sections were dewaxed in xylene, rehydrated through graded alcohols, stained with hematoxylin, differentiated in 1% acid alcohol for 30 s and counterstained with eosin. Masson staining used a trichrome staining kit (Solarbio, Beijing, China) and included Bouin’s solution at 56 °C for 1 h, Weigert’s iron hematoxylin for 10 min, Biebrich scarlet–acid fuchsin for 10–15 min, phosphomolybdic–phosphotungstic acid for 10–15 min, aniline blue for 5–10 min and 1% acetic acid for 2–5 min. Sections were dehydrated and cleared before mounting. Initial fixation, decalcification and section thickness were not documented.

### 2.3. Single-Cell Libraries and Stromal Analysis

Four ZMS libraries—SC1, DO1, SC3 and DO3—represented sham and distraction at days 1 and 3, with one biological sample per condition/day. Tissues were preserved in single-cell preservation solution (SeekGene, Beijing, China), washed in precooled RPMI1640, minced and digested at 37 °C with collagenase II (Sigma-Aldrich, St. Louis, MO, USA; V900892-100MG) and DNase I (Sigma-Aldrich, St. Louis, MO, USA; DN25-1G) until tissue dissociation. Enzyme concentrations and elapsed digestion time were not documented. Digests passed through 70 μm filters and were centrifuged at 300× *g* and 4 °C for 5 min. Pellets were resuspended in 1 mL RPMI1640/2% FBS and treated with red-cell lysis solution (Solarbio, Beijing, China; R1010). AO/PI staining and analysis using a Countstar Rigel S2 analyzer (Countstar, Shanghai, China) assessed cell quality; actual viability/nucleated-cell values and whether conditional dead-cell/debris removal was performed were unverified. After two RPMI1640 washes, cells were resuspended at 1 × 10^6^ cells/mL in PBS containing 0.04% BSA. SeekOne MM Single Cell 3′ libraries (SeekGene, Beijing, China; SO01V3.1) underwent SPRI purification and qPCR quantification (KAPA Biosystems, Wilmington, MA, USA; KK4824), followed by NovaSeq 6000 PE150 sequencing (Illumina, San Diego, CA, USA). The documented atlas contained 29,295 cells (OMIX005296).

Archived mouse gene-count matrices for SC1, DO1, SC3 and DO3 are supplied as Supplementary Single-Cell Source Data, with 10,895, 10,194, 12,627 and 9689 input barcodes, respectively, and 32,285 features per matrix. These 43,405 input barcodes are not the final 29,295-cell atlas subset. The original cell-selection metadata and panel-generating objects were not recovered. Input-file checks were performed, but the displayed integration, annotation and trajectory analyses were not rerun.

The documented workflow used Seurat 4.3.0 in R 4.2.2 to integrate the four libraries and subcluster fibroblast-like stromal cells. Principal component analysis (PCA) preceded UMAP visualization. The methods narrative describes CCA-based integration, whereas the appendix records SCT-based integration using 3000 features and 30 principal components for the atlas. For stromal subclustering, the appendix records LogNormalize, 2000 variable features, 20 principal components and the first 10 components for UMAP. The narrative and appendix also differ in quality-control settings. These records establish that PCA was included in the workflow but do not verify every parameter used to generate the final displayed panels. Table S1 distinguishes the documented steps from unresolved execution details. Cluster labels are descriptive transcriptional annotations: *Spp1*-Fib and *Ptprb*-Fib do not define validated lineages. Cellular composition means the fraction of retained cells assigned to each annotation within one SC1, DO1, SC3 or DO3 library. These fractions describe the captured cells and do not establish replicated differences in tissue cell abundance.

Monocle2 version 2.26.0 was used to construct a DDRTree embedding and order stromal cells. The displays show cluster identity, relative pseudotime, computational state and selected transcript expression on the same projection. Pseudotime represents relative transcriptional ordering, not elapsed time, anatomical position or lineage tracing. *Creb3l1* overlays concern transcript expression and do not measure CREB3L1 regulon activity. One biological sample per condition and day precludes a replicated test of distraction effects from these displays.

### 2.4. Extended Enrichment and Intercellular Communication

Supplementary enrichment displays comprise Gene Ontology categories, KEGG over-representation plots where indicated, and protein-association networks for neutrophil, monocyte and stromal gene sets. The stromal supplementary dot plot shows Gene Ontology molecular functions. These existing displays were retained for descriptive context. Complete input gene lists, enrichment backgrounds and final analysis outputs were unavailable, so the enrichment calculations were not reproduced in this revision.

CellChat infers potential communication from expression-compatible ligand–receptor relationships [9]. The documented intercellular-communication workflow used CellChat 1.6.1, CellChatDB.mouse, secreted-signaling interactions and PPI.mouse. The appendix records a minimum-cell filter of 10, pathway-probability calculation and network aggregation. The retained panels display global interaction counts and SPP1 or CXCL pathway predictions alongside ligand/receptor transcript distributions. MPCs is an undefined label in the original annotations and is retained without assigning a new lineage identity. Network edges denote expression-based predicted interactions; they do not measure ligand secretion, physical contact or signaling activity.

### 2.5. Culture Establishment and Characterization

ZMS tissue was obtained from six 4-week-old male C57BL/6J donor mice. Surrounding excess bone, muscle and periosteal tissue were removed, and explants were washed with sterile phosphate-buffered saline. Explants were distributed in 6 cm dishes containing 9 mL complete high-glucose DMEM supplemented with fetal bovine serum (Gibco, Grand Island, NY, USA) and 1% penicillin–streptomycin (HyClone, Logan, UT, USA), and incubated at 37 °C and 5% CO_2_. A further 5 mL of fresh complete medium was added after 6 h; outgrowing adherent cells were passaged with 0.25% trypsin at approximately 80% confluence. Passages 3–7 were used. During revision, the author clarified that the reported qPCR experiment used tissues pooled from six donor mice to establish one cell preparation. The exact passage used for each endpoint and the vessel-level links between the separate characterization, immunostaining and qPCR assays remain undocumented. Passage-dependent phenotype stability was not assessed. We therefore refer to this material as expanded suture-derived stromal cells (SDSCs), without assuming equivalence to freshly isolated suture populations.

Culture characterization used approximately 21-day osteogenic, adipogenic and chondrogenic induction protocols using differentiation kits (Haixing Biosciences, Suzhou, China). Osteogenic and adipogenic cultures were seeded at 2 × 10^4^ cells/cm^2^, and chondrogenic induction used pellets of approximately 3 × 10^5^ cells. Following fixation, cultures were assessed by Alizarin Red S, Oil Red O or Alcian Blue staining, respectively. Flow cytometry examined CD90, CD44, Sca-1, CD29, CD31, CD34 and CD117 using a mouse mesenchymal-cell marker kit (Cyagen, Guangzhou, China) and a MoFlo XDP instrument (Beckman Coulter, Brea, CA, USA). The protocol included negative-control and compensation tubes. Displayed percentages refer to the gates shown in the original plots. Raw FCS files, full gating records, matched non-induced images and independent culture-level replicate records were unavailable. These assays characterize the expanded material; they are not tests of phenotype preservation across passages or outcomes of stretch or siRNA treatment.

### 2.6. Cyclic Stretch and siRNA Groups

Cells were seeded at 2 × 10^5^ cells per elastic chamber coated with 2% type I rat-tail collagen. At approximately 80% confluence, a Cell Tank system (Dongdi, Beijing, China) applied 10% elongation at 1 Hz for 6 h/day over 3 days. Cultures were collected after the final loading session, with static cultures as the unloaded comparator. The presented culture comparison uses this loading regimen and the 3-day endpoint; no verified dose-response series or adequately mapped response time course was available for analysis.

Cy3-labeled mouse *Creb3l1*-targeting siRNAs and an accompanying negative control were supplied by Beijing Mijia (Beijing, China) and purchased through Beijing Yuanbohui (Beijing, China). One targeting siRNA was selected after screening three candidates, but raw measurements establishing knockdown efficiency were unavailable. Cells received 50 nM targeting or negative-control siRNA using Lipofectamine 2000 (Invitrogen, Carlsbad, CA, USA). Reagent and siRNA were separately diluted in Opti-MEM (Gibco, Grand Island, NY, USA). After 5 min of reagent incubation, the preparations were combined for 20 min at room temperature and added to cultures. Cells were cultured for 48 h before reseeding into imaging dishes or stretch chambers. In the qualitative immunofluorescence sets (Figure S2), SC denotes static negative-control-siRNA culture, si-*Creb3l1* denotes static targeting-siRNA culture, DO3 denotes 3-day stretch with negative-control siRNA, and DO3-si denotes 3-day stretch with targeting siRNA. Quantitative culture data are restricted to SC and DO3 under negative-control siRNA. Author clarification during revision established that SC1 and SC2 were two separate static culture dishes, and 3D-1, 3D-2 and 3D-3 were three separately treated stretch dishes/chambers. Each vessel was harvested for a separate RNA extraction. All five vessels came from the same pooled six-donor preparation and were studied in one experiment. Each target was measured in two qPCR wells per RNA sample. No one-to-one matched pairs or independent donor-preparation replicates were established; identical suffixes were not used to assign pairs.

### 2.7. qPCR, Immunostaining and Figure Preparation

RNA was extracted using a TRIzol-based procedure and reverse-transcribed. The recorded qPCR protocol used 20 μL SYBR Green reactions, 95 °C for 3 min, 40 cycles of 95 °C for 5 s and 60 °C for 15 s, and melting-curve analysis. Expression was normalized to *Gapdh* by the comparative-Cq method. Primer sequences are provided in Table S2. Throughout the main text, figures, tables and Supplementary Materials, mouse genes/transcripts are written in italics (e.g., *Creb3l1* and *Spp1*), whereas protein names are written in roman type (CREB3L1 and osteopontin, OPN). The original qPCR assay label “CREB” maps to *Creb3l1*, and “Opn”/”OPN” maps to *Spp1*; source labels are preserved in raw records and provenance documentation. CellChat pathway names (e.g., SPP1) and protein-association network identifiers are retained as database labels in roman type. Gene symbols within editable cell annotations follow the transcript convention. Labels retained within archived raster or outlined artwork are preserved as source labels and explained in the relevant figure legends; published reference titles retain their source nomenclature. Technical wells are measurements within an animal or culture-vessel RNA sample, not additional experimental units.

A rabbit polyclonal anti-CREB3L1/OASIS antibody (Proteintech, Rosemont, IL, USA; catalog no. 11235-2-AP) was used. For immunohistochemistry, sections underwent deparaffinization and EDTA antigen retrieval at pH 8.0 (Solarbio, Beijing, China) for 15 min, treatment with 3% hydrogen peroxide for 25 min and blocking with 3% bovine serum albumin for 30 min. Primary-antibody incubation was at 4 °C overnight, followed by secondary antibody for 1 h, DAB development and hematoxylin counterstaining. The primary-antibody dilution used for tissue IHC could not be verified from the available record. For cultured-cell immunofluorescence, cells were fixed with 4% paraformaldehyde for 15 min and treated with 0.2% Triton X-100 for 30 min. After blocking with 1% bovine serum albumin for 1 h, cells received primary antibody at 1:200 and 4 °C overnight. Anti-rabbit Alexa Fluor 488 secondary antibody (Cell Signaling Technology, Danvers, MA, USA) was applied for 1.5 h, followed by DAPI (Solarbio, Beijing, China) for 15 min. Tissue and culture staining were interpreted qualitatively. The author clarified that the four displayed tissue IHC fields correspond to four different mice: one SC and one DO mouse at each of days 1 and 3. Each condition/day therefore has one animal, with no within-condition biological replication for staining inference. Exact animal identifiers and slide/scan links were not recovered. Validated quantitative measurements of total or nuclear CREB3L1 and independent culture-to-IF-field mappings remain unavailable.

Native image objects and vector plots from the original TIFF and PDF-compatible Illustrator files were used for figure assembly. Existing single-cell plots were retained, with revised labels explaining their grouping; they were not regenerated from expression matrices. Tissue immunohistochemistry was enlarged from the original high-resolution image objects. The two original culture immunofluorescence sets are displayed in Figure S2. Microscopy intensity, contrast and channels were unchanged, and no scientific values were inferred from plot geometry. Full source materials were retained. Native slide and confocal files were inspected for provenance. The four-mouse IHC assignment was clarified retrospectively by the author; exact animal/slide/scan links and independent culture-to-IF-field mappings were not recovered. Calculations were checked against raw instrument exports and assembled figure content was checked against supplied source panels. The authors remain responsible for the scientific interpretation. Manufacturers and catalogue details for some generic reagents, including RPMI1640, DMEM, bovine serum albumin, AO/PI stain and SPRI purification reagents, were not specified in the archived records and could not be retrospectively verified.

### 2.8. qPCR Calculations and Exploratory Statistics

Tissue qPCR calculations used four instrument exports. The day-1 and day-3 plates dated 9 January 2024 supplied *Spp1* and *Creb3l1*; the combined day-1/day-3 plate dated 21 January 2024 supplied *Osm*, *Lifr* and *Osmr*; and the day-7 plate dated 2 August 2023 supplied *Sp7*. Each distinct tissue sample code denotes a different animal. Culture *Creb3l1* and *Spp1* calculations used SC and 3D codes from the cell plate dated 21 January 2024, denoting static and 3-day stretched negative-control-siRNA cultures. For each target and sample within a plate, finite technical-well Cq values were averaged arithmetically. ΔCq was the target mean minus the corresponding *Gapdh* mean. ΔΔCq was the sample ΔCq minus the mean ΔCq of valid controls for that target and plate, with tissue controls matched by harvest day. Relative expression was 2^−ΔΔCq^, and the plotted log2 relative expression equals −ΔΔCq. Plots show each animal or culture vessel with arithmetic mean ± sample SD on this log2 scale. Culture dispersion concerns vessels from the same preparation and experiment. The control mean is zero by construction, but individual control variation is retained. Samples were not pooled across plates or treated as longitudinal observations. Earlier plates also contained sparse, sometimes high-Cq *Osm* measurements; these were not combined with the selected 21 January run or used to replace its missing values. Original_qPCR_Exports.zip supplies all eight archived instrument exports, including records outside the selected analyses. Separate calibration does not support absolute comparisons across days or between tissue and culture. Supplementary Data includes raw wells, sample calculations, descriptive summaries and quality-control flags.

*Spp1* and *Creb3l1* tissue measurements included four animals in each of SC1, DO1, SC3 and DO3. *Sp7* included two SC7 and five DO7 animals. These plates contained three finite target and three finite *Gapdh* wells per animal. Culture qPCR included two SC dishes and three DO3 stretch chambers, with two target and two *Gapdh* wells per vessel-derived RNA sample. The *Osm*/*Lifr*/*Osmr* plate recorded three animals per group with two wells per target. On the selected 21 January plate, *Osm* had no finite target Cq for SC1-4 or SC3-2, yielding two, three, two and three available animals in SC1, DO1, SC3 and DO3, respectively. *Osm* values for SC1-3 and DO1-1 each used one finite target well. *Lifr* and *Osmr* had three animals per group. Missing values were not imputed or replaced with a fixed Cq. All finite wells in the selected source records were retained. Cq ≥ 35, technical-well ranges of >0.5 to ≤1 cycle or >1 cycle, and single-well observations were flagged descriptively rather than used as exclusion criteria. The comparative-Cq calculation assumes comparable amplification efficiencies. The instrument exports do not establish amplification efficiency, no-template-control performance or melt-curve specificity.

Animal-level tissue qPCR was evaluated using two-sided exact Mann–Whitney tests comparing distraction with same-day sham controls on the log2 relative-expression scale. All 11 available target-by-day comparisons formed one multiplicity family: *Creb3l1*, *Spp1*, *Osm*, *Lifr* and *Osmr* at days 1 and 3, and *Sp7* at day 7. Holm-adjusted *p* values are the primary inferential results; unadjusted values are supplied for transparency in Table S3 and the Exact_Statistics worksheet. Tests were added as exploratory analyses during revision and were not prespecified confirmatory endpoints. The rank test evaluates differences in distributions/rank ordering; the distraction-minus-sham mean log2 difference is a separate descriptive effect estimate, not the parameter tested by Mann–Whitney. Statistical significance was assessed at a two-sided Holm-adjusted *p* < 0.05. Small groups yield discrete *p* values and limited power, and nonsignificance does not establish absence of an effect. With these sample sizes, the smallest attainable two-sided exact *p* value is 2/70 = 0.0286, which exceeds the first Holm threshold of 0.05/11 = 0.00455; therefore, none of the 11 comparisons can attain family-wise significance at 0.05 under this analysis, even with complete rank separation. SD describes the observed dispersion, not a confidence interval. After clarification of the culture-vessel units, two-sided exact Mann–Whitney tests were additionally calculated for the two culture targets, with Holm adjustment within a separate two-target culture family (Table S4). The culture and tissue families address different experimental systems. Culture tests assume independent, exchangeable vessel-level measurements under the null; random allocation was not documented. The inference is restricted to the single pooled preparation and experiment and cannot estimate variation between donor preparations or experiments. No paired or equivalence test was performed. With two versus three vessels, the smallest attainable two-sided exact *p* value is 0.200, so conventional significance is unattainable even with complete rank separation. Table S4 also provides descriptive effects and marginal exact 95% location-shift intervals, which are unbounded at these culture sample sizes. These analyses were exploratory additions during revision. Single-cell plots, enrichment displays and communication networks were not treated as replicated sample-level tests. To describe uncertainty, Table S3 also reports Hodges–Lehmann location shifts (medians of all cross-group DO–SC differences) and marginal equal-tailed exact rank-inversion intervals under an independent, continuous common-shape location-shift model. These intervals have at least 95% coverage, are not adjusted for multiplicity and are not intervals for the mean differences. Discreteness yields 97.142857% coverage for the four 4-versus-4 comparisons; the remaining seven intervals are unbounded at the 95% level.

**Figure 1:**
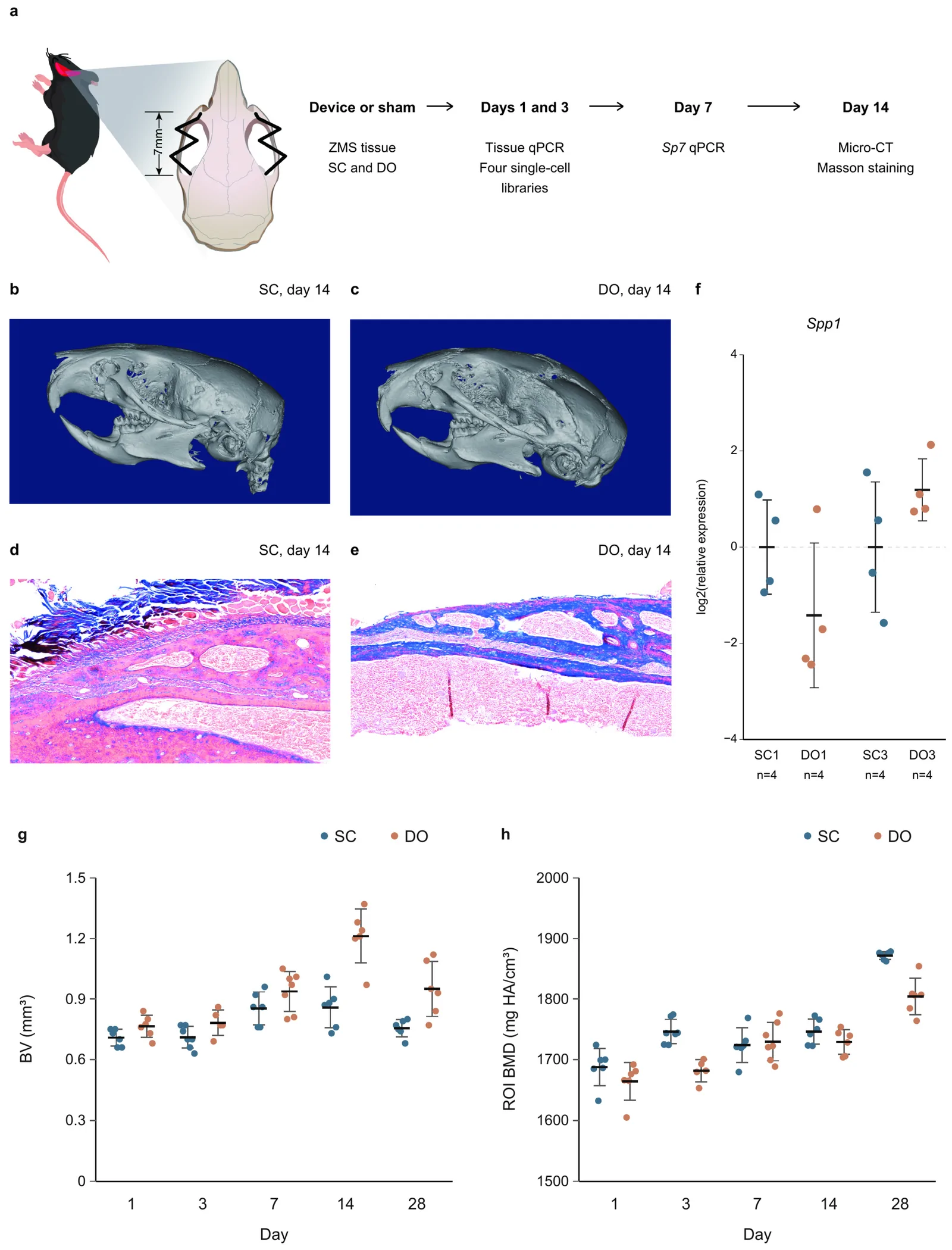
Sutural morphology and *Spp1* expression. (**a**) Distraction device and selected sampling landmarks; the day-14 micro-CT label identifies the displayed reconstructions, whereas quantitative measurements in (**g**,**h**) cover five time points. The complete tissue, single-cell and culture design is shown in Scheme 1. (**b**,**c**) Day-14 lateral micro-CT reconstructions from sham (SC, (**b**)) and distraction (DO, (**c**)) groups. (**d**,**e**) Day-14 Masson-stained SC (**d**) and DO (**e**) sections. Images provide qualitative anatomical and matrix-staining context. The experimental record specifies a 50 μm scale for Masson staining, but a calibrated scale bar is unavailable in the original composite. (**f**) Tissue *Spp1* qPCR at days 1 and 3. Points represent four different animals in each of SC1, DO1, SC3 and DO3; central marks and error bars show mean ± SD of log2 relative expression (−ΔΔCq). Three technical wells per target and animal were averaged, normalized to *Gapdh* and calibrated to the same-day SC mean ΔCq. Control means are zero by construction, while individual variation is retained. Two-sided exact Mann–Whitney *p* = 0.200 for each day; Holm-adjusted *p* = 1.000 for each day across all 11 tissue comparisons. Source values, quality-control flags and tests are in Supplementary Data and Table S3. Separate day-specific calibration does not support absolute cross-day expression comparisons. Tissue images and qPCR belong to the broader research program disclosed in Methods and are not an independent replication of the related publications. (**g**,**h**) Animal-level bilateral means for BV ((**g**), mm^3^) and regional BMD ((**h**), mg HA/cm^3^) at days 1, 3, 7, 14 and 28. SC/DO counts are 6/6, 7/5, 6/7, 6/6 and 6/6, respectively; dots show individual animals and central marks/whiskers show mean ± SD. The two ZMS-DO1-3 side records contribute one averaged point. Exact two-sided tests with ties were Holm-adjusted across the 10 micro-CT comparisons: adjusted *p* = 0.0346 for BV at day 14, 0.0227 for BMD at day 3 and 0.0216 for BMD at day 28; all other adjusted *p* values exceed 0.05. Full effects, uncertainty and provenance are in Table S5 and MicroCT_Source_Data.zip. These archived measurements include later same-condition animals from the same research program; batch identities, exact ROI dimensions, numeric segmentation threshold and original calibration measurements remain unavailable. Blue and orange points denote SC and DO, respectively (**f**–**h**).

Micro-CT BV and regional BMD were analyzed as a separate exploratory family of 10 same-day SC–DO comparisons across five time points. Two-sided exact Mann–Whitney statistics were evaluated by enumerating all group-label allocations and comparing the absolute distance of U from its null center; this calculation retains observed ties. Holm correction included all 10 micro-CT tests (two endpoints at five time points), with no comparisons dropped from the family. Table S5 reports all 10 raw and adjusted *p* values, including the seven comparisons that were not significant after adjustment. Figure 1g,h shows animal-level values and mean ± sample SD. Mean differences and arithmetic-mean ratios are descriptive effects. Table S5 also reports Hodges–Lehmann shifts and marginal 95% common-shape additive-shift confidence intervals obtained by inverting the same tie-aware permutation test; these are not confidence intervals for mean differences or ratios and are not adjusted for multiplicity. The accepted shift sets were connected for all 10 comparisons. No treatment-by-time interaction or batch-adjusted effect was estimated. These analyses concern archived measurements and do not constitute new experiments or independent replication of the broader study.

## 3. Results

### 3.1. Tissue Morphology and Exploratory Spp1 Measurements

The W-shaped device directed force across the zygomaticomaxillary complex (Figure 1a). The study design separates early molecular sampling at days 1 and 3, *Sp7* qPCR at day 7 and the main structural comparison at day 14 (Scheme 1). Day-14 micro-CT reconstructions document the location and appearance of the region, and Masson sections show blue-stained matrix within the sutural tissue (Figure 1b–e). Additional H&E fields depict the fibrous interface and adjacent bone across the 1–28-day interval (Figure S1). These qualitative observations provide anatomical context for the separate transcript measurements.

Reanalysis of the archived micro-CT measurements included 61 animals, with bilateral means used as the unit of analysis (Figure 1g,h; Table S5). The descriptive DO–SC mean BV difference at day 14 was +0.353 mm^3^ (exact *p* = 0.00433; Holm-adjusted *p* = 0.0346). Regional BMD mean differences were −64.681 mg HA/cm^3^ at day 3 and −67.739 mg HA/cm^3^ at day 28 (adjusted *p* = 0.0227 and 0.0216, respectively). The other seven comparisons were not significant after correction across all 10 tests. These measurements provide structural context; incomplete ROI, calibration and batch records limit reproducibility and interpretation, and the findings do not establish a CREB3L1-dependent remodeling mechanism.

The observed distraction-minus-sham mean differences in tissue *Spp1* log2 relative expression were −1.42 on day 1 and +1.19 on day 3, with four animals per group. Distraction-group means ± SD were −1.42 ± 1.51 and 1.19 ± 0.64, respectively, after same-day sham calibration (Figure 1f). Neither comparison was statistically significant: exact two-sided *p* = 0.200 for both days and Holm-adjusted *p* = 1.000 for both within the 11-comparison family (Table S3). These directional estimates describe the available animals with substantial uncertainty; they do not establish a temporal reversal of the underlying response. Tissue qPCR and day-14 staining concern separate harvests.

### 3.2. The Early Libraries Contain Heterogeneous Stromal Expression Profiles

**Figure 2:**
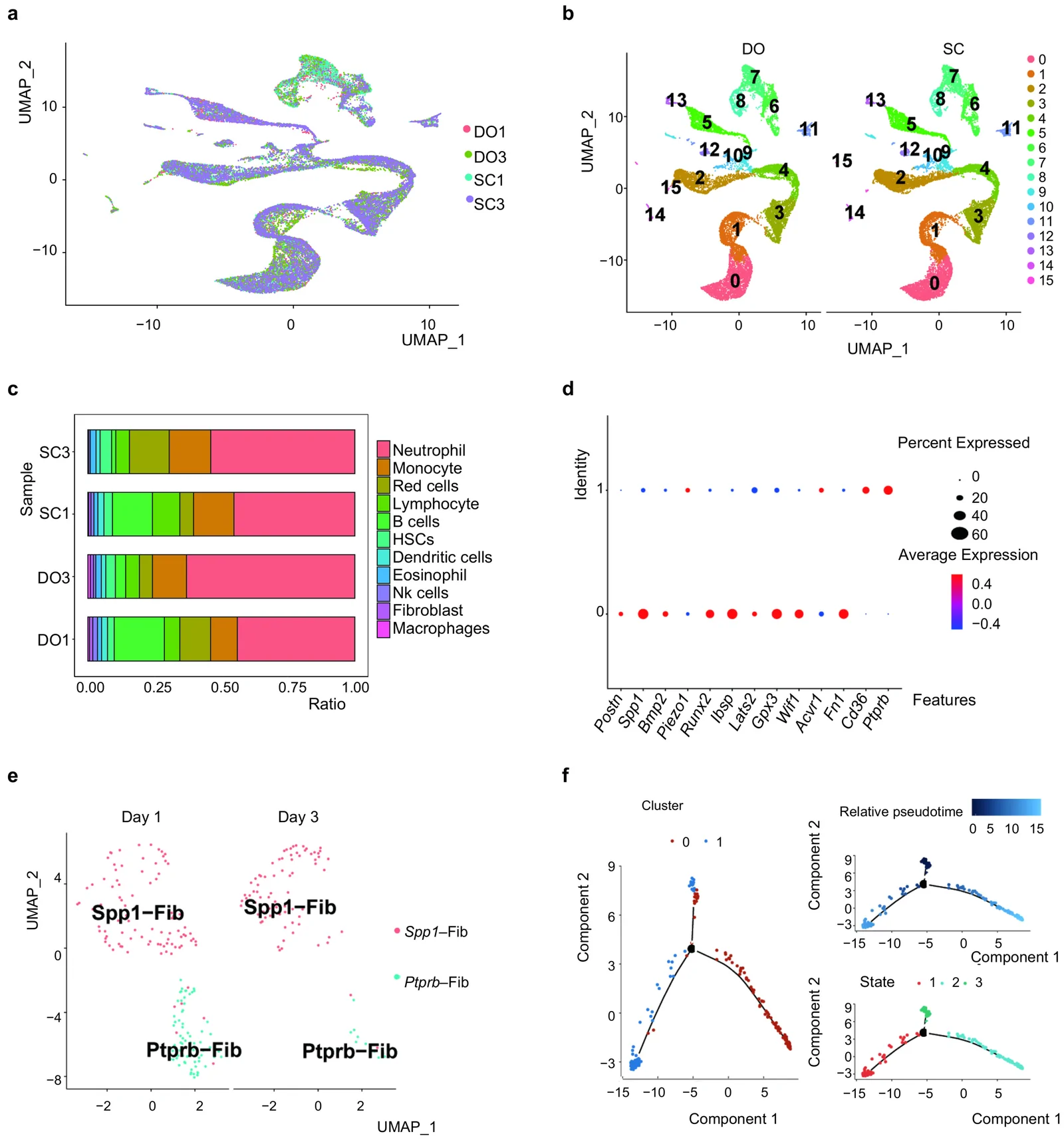
Cellular composition and stromal transcriptional profiles in four early ZMS libraries. (**a**) Integrated UMAP colored by the four sequencing libraries: SC1, sham day 1; DO1, distraction day 1; SC3, sham day 3; DO3, distraction day 3. (**b**) The 16-cluster atlas split by SC and DO, pooling days 1 and 3 within each condition. (**c**) Fraction of captured cells assigned to each original annotation within each library. HSCs is a legacy hematopoietic stem-cell annotation and does not establish functional stem-cell identity. (**d**) Selected stromal transcripts: dot size indicates percentage of expressing cells, and color indicates scaled average expression. Each library represents one biological sample; the panels are descriptive. Numerical-cluster labels in b are the archived expression-based annotations: 0, 1, 3 and 4, neutrophil; 2, monocyte; 5 and 13, red cells; 6, lymphocyte; 7 and 8, B cells; 9, hemopoietic stem cells; 10, dendritic cells; 11, eosinophil; 12, NK cells; 14, fibroblast; and 15, macrophages. The stem-cell label does not establish functional stemness. (**e**) Day-1 and day-3 stromal UMAPs, pooling SC and DO within each day; *Spp1*-Fib and *Ptprb*-Fib are expression-based annotations. The source annotation strings “*Spp1*–Fib” and “*Ptprb*–Fib” embedded in the archived scatter panels are retained and denote *Spp1*-expressing and *Ptprb*-expressing fibroblast-like cells, respectively. Editable gene labels follow the italic transcript convention in Section 2.7. (**f**) The same stromal DDRTree projection colored by cluster, relative pseudotime and computational state. PCA was documented before UMAP, as explained in Methods; the plots shown here are UMAP and DDRTree displays rather than PCA scatterplots. Existing outputs were retained without reanalysis. Each library represents one biological sample, so cells are not biological replicates and neither composition nor embedding establishes a replicated SC–DO difference or lineage transition.

The integrated UMAP and composition plots summarize the cells captured in SC1, DO1, SC3 and DO3 (Figure 2a–c). Here, library-specific composition means the fraction of retained cells assigned to each annotated population within one sequenced sample. Immune compartments and fibroblast-like stromal cells were represented in the displays. The condition-split atlas pools days 1 and 3 within SC and DO, whereas the composition bars retain the four separate libraries. Each condition and harvest day has only one biological sample; these plots therefore describe the captured material and do not test a reproducible distraction-related shift in cell abundance.

Stromal subclustering resolved two transcriptional profiles. Cluster 0 displayed prominent *Spp1*, *Bmp2*, *Runx2*, *Ibsp* and *Gpx3* signals, whereas *Cd36* and *Ptprb* were more prominent in cluster 1 (Figure 2d). Both profiles, designated *Spp1*-Fib and *Ptprb*-Fib, appeared in the day-specific embeddings, which pool SC and DO cells within each day (Figure 2e). *Postn*, *Piezo1*, *Lats2*, *Wif1*, *Acvr1* and *Fn1* provided additional marker context. These annotations distinguish sampled expression profiles without establishing homogeneous cell identities or treatment effects.

The stromal cells occupied a branched Monocle2/DDRTree embedding shown by cluster, relative pseudotime and computational state (Figure 2f). These views provide a common transcriptional coordinate system for the subsequent gene overlays. Their ordering does not establish a lineage transition, an anatomical location or a differentiation rate.

### 3.3. CREB3L1 Expression in Stromal Profiles and Tissue

**Figure 3:**
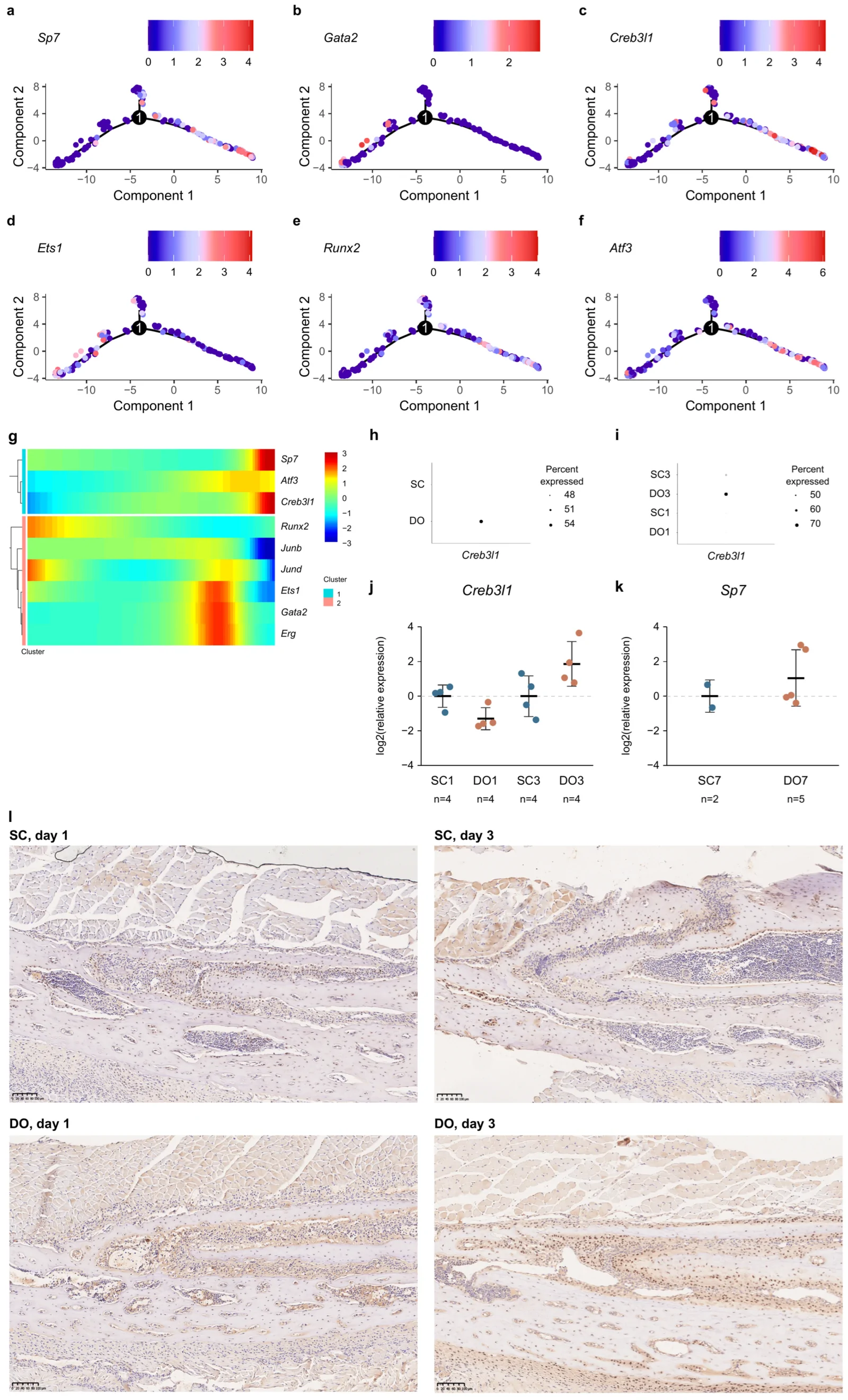
CREB3L1-associated expression patterns and tissue observations. (**a**–**f**) Stromal DDRTree overlays for *Sp7* (**a**), *Gata2* (**b**), *Creb3l1* (**c**), *Ets1* (**d**), *Runx2* (**e**) and *Atf3* (**f**). Gene-specific scales are not comparable across genes. The circled “1” identifies the branch point in the displayed DDRTree trajectory. (**g**) Nine-transcript heatmap across relative pseudotime. These displays measure transcript distribution, not regulon activity. (**h**,**i**) *Creb3l1* detection pooled by condition across days (**h**) and separated into SC3, DO3, SC1 and DO1 (**i**). Dot size follows percentage-expressed keys; shading is not a calibrated intensity scale. Both panels describe the same four libraries. (**j**) Tissue *Creb3l1* qPCR at days 1 and 3, with four animals per group. (**k**) Tissue *Sp7* qPCR at day 7, with two SC7 and five DO7 animals. Points denote animals; central marks and error bars show mean ± SD of log2 relative expression. Three technical wells per target and animal were averaged, normalized to *Gapdh* and calibrated to same-day SC. Exact two-sided Mann–Whitney *p* values for *Creb3l1* day 1, *Creb3l1* day 3 and *Sp7* day 7 are 0.057, 0.114 and 0.571; corresponding Holm-adjusted values across all 11 tissue comparisons are 0.629, 1.000 and 1.000. Calculations and quality-control flags are in Supplementary Data and Table S3. Each single-cell condition and day has one biological sample; the existing displays were not recalculated. (**l**) Enlarged CREB3L1 tissue immunohistochemistry from SC and DO at days 1 and 3, with DAB (brown) and hematoxylin (blue-purple). The author confirmed that the four fields correspond to four different mice, one per condition/day; days were not pooled as biological replicates. Exact animal/slide/scan identifiers remain unavailable. Original image pixels, group/day labels and scale marks are retained. The fields are qualitative and do not quantify protein abundance or nuclear activation. Blue and orange points in j,k denote SC and DO, respectively.

Six transcript overlays mapped *Sp7*, *Gata2*, *Creb3l1*, *Ets1*, *Runx2* and *Atf3* onto the same stromal DDRTree projection (Figure 3a–f). *Creb3l1* and *Sp7* signals were apparent toward the right-hand branch (positive Component 1), whereas *Gata2* and *Ets1* signals were more evident toward the lower-left branch. These locations are coordinates in the displayed transcriptional embedding, not anatomical regions or evidence of a developmental direction. *Runx2* and *Atf3* supplied additional expression distributions. The heatmap placed these genes alongside *Junb*, *Jund* and *Erg* (Figure 3g). Gene-specific color ranges do not permit comparisons of absolute expression intensity across different gene overlays.

The *Creb3l1* dot plot pooled across days displayed a larger percentage-expressed marker in distraction-labeled stromal cells (Figure 3h). Separating the same cells by library showed the largest marker in DO3 (Figure 3i). These are descriptive detection fractions within the four sampled libraries. The pooled view does not add independent observations, and neither view establishes a statistically replicated increase in *Creb3l1* expression.

Tissue *Creb3l1* mean log2 relative expression was −1.30 ± 0.64 in DO1 and 1.85 ± 1.28 in DO3 after same-day sham calibration, with four animals per group (Figure 3j). The respective distraction-minus-sham mean differences were −1.30 and +1.85; exact *p* values were 0.057 and 0.114, and Holm-adjusted values were 0.629 and 1.000. Day-7 *Sp7* values were 0.00 ± 0.93 in SC7 and 1.05 ± 1.62 in DO7, with two and five animals, respectively (Figure 3k; exact *p* = 0.571, Holm-adjusted *p* = 1.000). None of these comparisons was statistically significant (Table S3). Day-7 *Sp7* is a separate observation, not an early time course. Brown DAB staining indicated detectable CREB3L1 immunoreactivity in the illustrated sham and distraction ZMS sections at days 1 and 3 (Figure 3l). Protein abundance and nuclear activation were not quantified. Tissue qPCR includes multiple cell populations, and neither qPCR nor total immunoreactivity assigns the signal specifically to stromal cells or establishes CREB3L1 cleavage or nuclear translocation.

### 3.4. Expanded Cultures Show Stromal Markers and Induction-Associated Staining

**Figure 4:**
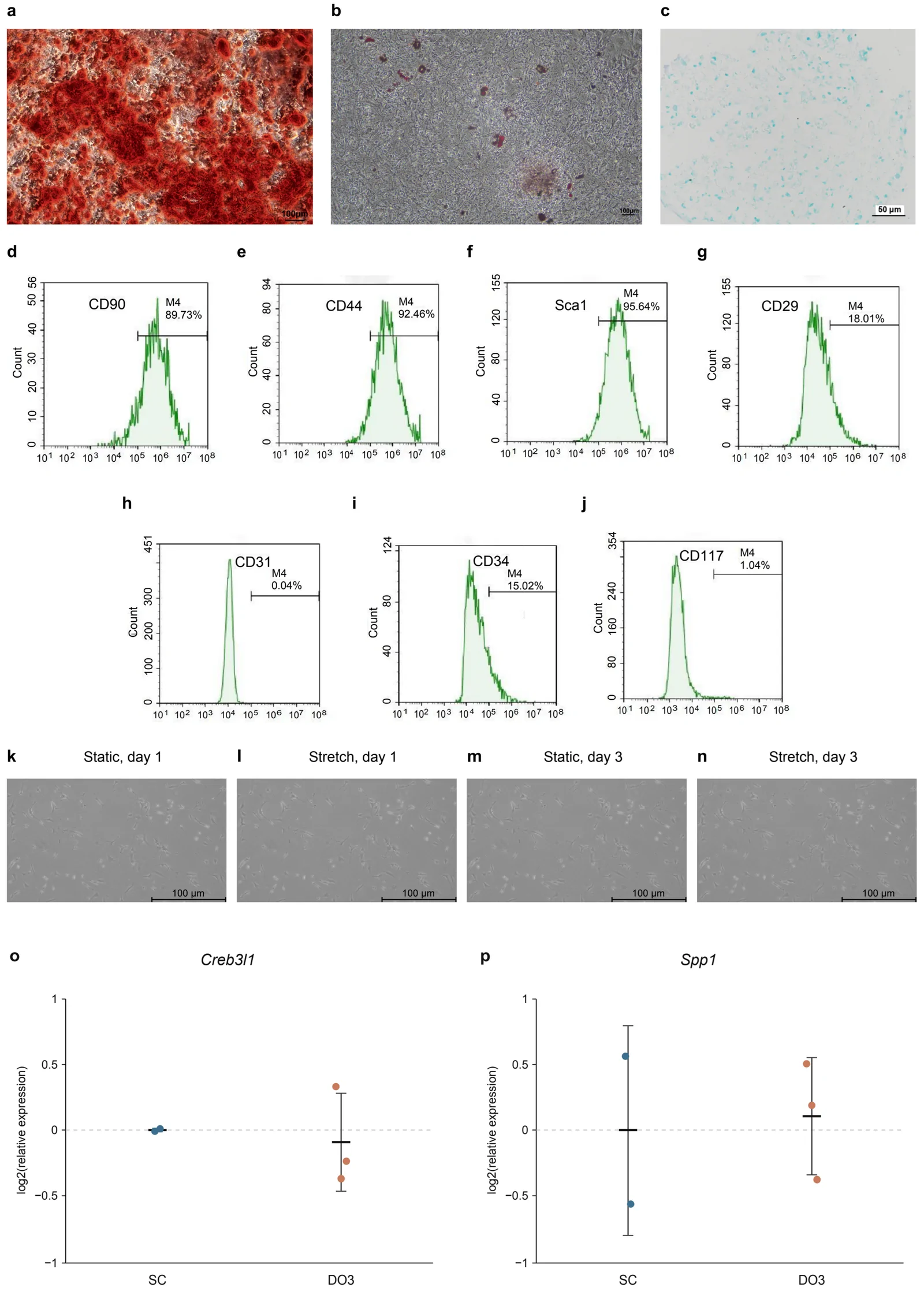
Characterization and transcript measurements of expanded suture-derived stromal cultures. (**a**–**c**) Expanded ZMS-derived stromal cultures after approximately 21 days of osteogenic, adipogenic and chondrogenic induction, assessed with Alizarin Red S (**a**), Oil Red O (**b**) and Alcian Blue in an induced cell-pellet section (**c**). Source scale labels, 100 μm (**a**,**b**) and 50 μm (**c**). The label in c was corrected against the original image and bar geometry; the label in b is retained from the original composite without independent calibration. These qualitative characterization assays were separate from the stretch/siRNA experiments. Matched non-induced images and independent culture-level replicate records were unavailable. (**d**–**j**) Surface-marker flow-cytometry histograms, retaining percentages within the displayed gates: CD90, 89.73% (**d**); CD44, 92.46% (**e**); Sca-1, 95.64% (**f**); CD29, 18.01% (**g**); CD31, 0.04% (**h**); CD34, 15.02% (**i**); and CD117, 1.04% (**j**). Raw FCS files, full gating records and independent culture-level replicate information were unavailable for reanalysis. The profiles characterize the expanded population without establishing homogeneous stem-cell identity or phenotype stability across passages. (**k**–**n**) Phase-contrast images of static and stretched cultures at day 1 (**k**,**l**) and day 3 (**m**,**n**). SC and DO denote static and stretched culture, respectively. Scale bars, 100 μm. These images describe appearance; proliferation, viability, orientation and differentiation were not quantified from them. Microscopy intensity and contrast were unchanged during assembly. (**o**,**p**) *Creb3l1* (**o**) and *Spp1* (**p**) qPCR in static (SC) and stretched (DO3) cultures receiving negative-control siRNA. Stretch was 10% elongation, 1 Hz and 6 h/day for 3 days. Each point is one of two SC dishes or three DO3 chambers from one pooled six-mouse preparation in one experiment. Bars show mean ± SD across vessels on the log2 scale (−ΔΔCq). Two technical wells per target/RNA sample were averaged, normalized to *Gapdh* and calibrated to the SC mean ΔCq. Exact two-sided Mann–Whitney *p* = 0.800 and 1.000; both Holm-adjusted *p* values are 1.000 within the two-target family (Table S4). No independent donor-preparation replication, pairing, equivalence or targeting-siRNA effect is established. Source values and QC flags are supplied; qualitative immunofluorescence is in Figure S2. Blue and orange points in o,p denote static SC and stretched DO3 cultures, respectively.

Expanded cultures were characterized by induction-associated staining, surface-marker profiles and phase-contrast images (Figure 4). Alizarin Red S, Oil Red O and Alcian Blue staining were observed after the corresponding induction protocols (Figure 4a–c). These images document the characterized culture material. They were separate from the stretch and siRNA comparisons and do not show that the same phenotype was maintained across passages 3–7.

The displayed flow-cytometry gates contained 89.73% CD90-positive, 92.46% CD44-positive and 95.64% Sca-1-positive events. Fractions for CD29, CD31, CD34 and CD117 were 18.01%, 0.04%, 15.02% and 1.04%, respectively (Figure 4d–j). These profiles describe a mixed expanded population without establishing a homogeneous or lineage-defined stem-cell identity. Phase-contrast images showed adherent cells in static and stretched cultures at days 1 and 3 (Figure 4k–n). The characterization assays and images do not provide quantitative functional outcomes for stretch or siRNA treatment, or a direct comparison with freshly isolated cells.

### 3.5. Descriptive Transcript Measurements After Three Days of Culture Stretch

For cultures receiving negative-control siRNA, *Creb3l1* mean log2 relative expression in DO3 was −0.09 ± 0.37 and *Spp1* was 0.11 ± 0.45, relative to the respective SC means of zero. Two static dishes and three separately treated stretch chambers from one pooled preparation were available (Figure 4o,p; Supplementary Data). The corresponding mean differences were −0.09 and +0.11 log2 units. Exact two-sided culture-vessel *p* values were 0.800 for *Creb3l1* and 1.000 for *Spp1*; both Holm-adjusted values were 1.000 (Table S4). With two static dishes versus three stretch chambers, the smallest attainable two-sided exact *p* value is 0.200, even with complete rank separation. The SDs summarize vessel variation within one preparation and experiment; no donor-level replication or equivalence test was available. They do not establish a reproducible absence of a stretch response or a statistically supported difference from the tissue response.

The two original CREB3L1 immunofluorescence sets are presented as qualitative records in Figure S2. They show static negative-control and targeting-siRNA cultures, and static negative-control, stretched negative-control and stretched targeting-siRNA cultures. Fluorescence intensity was not quantified and protein suppression was not established. No raw targeting-siRNA qPCR measurements were available to quantify knockdown efficiency. The main culture qPCR comparison therefore addresses loading under negative-control treatment and supplies no estimate of a *Creb3l1*-targeting-siRNA effect.

### 3.6. Extended Immune Analyses Describe the Surrounding Transcriptional Environment

Neutrophil and monocyte gene-set displays included inflammatory, migratory and signaling-related categories. Stromal gene sets included matrix organization and ossification-related categories (Figures S3–S5). The six neutrophil annotations were further described by a marker heatmap, *Jun*/*Mpo*/*Mki67*/*Retnlg*/*Il1b*/*Ctse* expression overlays, within-library fractions and the pooled embedding (Figure S6). These complementary views describe heterogeneity within the immune compartment represented beside the stromal cells. They do not provide an additional dataset or biological replication of the library comparisons.

The SPP1 panels examined relative pathway information flow in DO3 and SC3 and global interaction-count networks in DO1 and SC1. They also included SPP1-focused networks with fibroblast-like cells as the displayed sender (Figure S7a–e). The focused four-population networks retained the source interaction counts for fibroblast-like cells, *Il1b*-Neu, *Jun*-Neu and the legacy MPCs label (Figure S7f,g). The paired violin plots placed prominent *Spp1* expression in fibroblast-like cells alongside *Cd44* and integrin-transcript expression in multiple annotated populations (Figure S7h). These expression distributions provide context for the predicted network edges without establishing physical cell interactions or a CREB3L1-mediated pathway.

The expression panels separated the cellular distributions of OSM-related transcripts. *Osm* signals were apparent within *Ctse*-Neu, *Jun*-Neu and the legacy MPCs annotation, *Lifr* was prominent in fibroblast-like cells, and *Il6st* appeared in multiple annotations (Figure S8a). Tissue *Osm* log2 relative-expression means were 1.85 ± 0.94 in DO1 and 2.27 ± 0.64 in DO3 relative to same-day sham means (Figure S8b). Available *Osm* animal counts were 2, 3, 2 and 3 in SC1, DO1, SC3 and DO3. Missing targets, single-well estimates and high Cq values limit interpretation. *Lifr* means were −0.93 ± 0.90 in DO1 and 1.45 ± 1.05 in DO3; *Osmr* means were 0.15 ± 0.50 and 1.05 ± 0.62, respectively, with three animals per group (Figure S8c,d). All six corresponding Holm-adjusted *p* values were 1.000 (Table S3). Across the complete family of 11 tissue comparisons, none was significant before or after adjustment. These findings neither establish OSM receptor activation nor place CREB3L1 downstream of OSM signaling.

The paired CXCL heatmaps and networks show predicted sender–receiver relationships in DO1 and SC1. The gene-level violins place *Cxcl12* prominently in fibroblast-like cells and show distinct distributions of *Cxcl2*, *Cxcl3*, *Cxcr2* and *Cxcr4* (Figure S8e–g). These panels broaden the immune and stromal context of the four libraries. Transcript detection and network predictions do not demonstrate receptor activation or downstream signaling.

## 4. Discussion

This study examined whether CREB3L1-associated expression could be described in the early response of distracted mouse ZMS tissue and in expanded suture-derived cultures exposed to stretch. The four single-cell libraries and tissue immunostaining supplied descriptive expression and localization information. Animal-level *Creb3l1* and *Spp1* estimates were negative relative to same-day sham references on day 1 and positive on day 3, but no tissue comparison was statistically significant after correction across all 11 tests. The small culture series yielded mean differences near zero without establishing either equivalence or a reproducible absence of response. These observations identify questions about tissue context and measurement timing; they do not demonstrate a common CREB3L1 mechanism across systems. The interpretation is consequently preliminary, with expression patterns considered separately from protein activation and functional matrix production.

Several differences could explain why the tissue and culture estimates were not similar. The nominal 30 g spring force in vivo is a force applied to a complex tissue geometry, whereas 10% cyclic elongation in vitro is an imposed chamber deformation. Without tissue-strain measurements, these exposures cannot be converted into matched doses. The culture regimen also imposed a frequency and daily duration that differ from the in vivo device, and no verified loading-dose series was available for the present analysis. Sampling after the final culture loading session may miss an earlier or delayed transcriptional response. In a separate mouse coronal-suture study, the proliferation response depended on both the number of loading sessions and the interval after loading [10]. That finding concerns a different suture and endpoint, but supports treating exposure and sampling time as variables to test rather than assuming matched kinetics here.

Cell composition and expansion provide additional explanations. Whole ZMS tissue contains immune, vascular and stromal components and retains a three-dimensional matrix, while the cultures are selected by explant outgrowth and passage on a collagen-coated substrate. Passages 3–7 may alter cellular state or mechanical responsiveness, but passage-dependent drift was not measured in this experiment. A recent study identified passage-associated senescence signatures in mesenchymal stromal/stem-cell datasets and validated selected findings in human umbilical-cord cultures [11]; it does not demonstrate dedifferentiation of the mouse cultures examined here. Our induction stains and surface-marker profiles cannot establish preservation of an in situ phenotype. The qPCR cells came from one preparation pooling six donor mice, and all five culture vessels were studied in one experiment. The two static dishes and three stretch chambers provide within-preparation vessel replication, but do not establish reproducibility across donor preparations or experiments. With these sample sizes, an undetected response remains plausible; the data should not be interpreted as equivalence or a confirmed negative biological result.

Force-responsive signaling provides several potential entry points. RhoA/ROCK–TAZ signaling has been implicated in calvarial distraction, while ROCK inhibition suppresses stretch-associated YAP nuclear localization in mesenchymal-cell cultures [12,13]. Liao et al. further showed that NF2 physically interacts with focal adhesion kinase (FAK) in cranial osteoblasts, supporting ERK1/2 and PI3K–AKT signaling and differentiation [14]. Mechanosensitive ion channels provide another route: a recent osteocyte study linked PIEZO1 to YAP–CCN–Src signaling, dendrite development and maturation [15]. These mechanisms establish ways for physical inputs to alter skeletal-cell state, but their operation is dependent on cell type and differentiation stage. *Piezo1* appears in our stromal marker display, yet transcript detection does not establish channel activity. In the collagen-coated stretch system, adhesion-dependent signaling, ion-channel responses and CREB3L1-associated transcription could operate in parallel. No direct FAK-, PIEZO1- or YAP-to-CREB3L1 connection was tested. In primary human dermal fibroblasts, substrate stiffening was linked to calcium influx, endoplasmic-reticulum stress and CREB3L1 processing and nuclear accumulation [16]. This skin-fibrosis setting provides a mechanistic precedent for testing a stiffness-associated response, but does not establish the same relationship in ZMS tissue.

Evidence from lineage-defined suture cells helps refine this question. Jing et al. found that conditional β-catenin deletion restricted *Gli1*-positive cell activation and remodeling during suture expansion [2]. In a facial-suture distraction model, Jin et al. implicated primary cilia and Hedgehog signaling in the osteogenic response of *Gli1*-positive cells [17]. These studies identify Wnt- and Hedgehog-dependent responses in experimentally defined populations, whereas our *Spp1*-Fib and *Ptprb*-Fib groups are transcriptional annotations. These developmental signals may establish a responsive cell state in which CREB3L1 contributes to matrix handling. Co-occurrence of osteogenic transcripts does not, however, establish a GLI1–CREB3L1–SP7 regulatory sequence. Distinguishing a shared cellular response from a serial pathway requires measuring CREB3L1 processing after a defined upstream perturbation, with a CREB3L1-specific rescue.

Intracellular adaptation may be as important as the initial force-sensing event. In cranial-suture expansion, Cai et al. linked PERK activation to TFEB nuclear translocation, followed by sequential mitochondrial biogenesis and mitophagy that supported organelle quality and osteogenic capacity [18]. In a different culture setting, Na et al. showed that matrix stiffness regulates mesenchymal-cell metabolism and mitochondrial dynamics through YAP during osteogenic differentiation [19]. Together, these studies connect mechanical context with the energy and organelle requirements of matrix production. CREB3L1 could coordinate an additional secretory response in this setting. However, these studies do not place CREB3L1 downstream of PERK–TFEB or establish that mitochondrial adaptation explains our findings. PERK phosphorylation, TFEB localization, metabolism and CREB3L1 cleavage would need to be assessed together to resolve whether these are coupled responses or parallel adaptations.

CREB3L1 abundance and activity need to be distinguished. In mouse dental papilla cells, immunofluorescence localized the amino-terminal form of CREB3L1 predominantly to nuclei after 12 and 24 h of differentiation induction [4]. A recent study of senescent fibroblasts in a cancer-stroma context also examined TGF-β1-associated CREB3L1 cleavage and collagen expression using immunoblotting and perturbation assays [20]. These observations demonstrate approaches for assessing processing and localization in defined cellular settings; they do not establish the same pathway in mechanically loaded sutures. In zebrafish, a mutant CREB3L1 fragment unable to enter the nucleus was associated with caudal-fin bifurcation defects [21]. Our *Bmp2* transcript display and total CREB3L1 immunoreactivity do not resolve these steps. Increased mRNA could accompany precursor accumulation without an increase in active nuclear protein.

Downstream of activation, matrix transcription and protein export can be experimentally separable. In CREB3L1-deficient odontoblast-lineage cells, restoring the target TMEM30B partially recovered extracellular protein levels without restoring DMP1/DSPP expression or mineralized-nodule formation [4]. This finding supports considering multiple components of matrix production and cautions against treating a transcript readout as sufficient evidence of osteogenesis. Dzamukova et al. linked mechanical loading in adolescent long bones to a PIEZO1–FAM20C–DMP1 pathway coupling matrix mineralization with vascular maturation [22]. Although that setting differs from ZMS distraction, it illustrates why secreted matrix proteins have consequences not captured by transcript abundance alone. A study of oral squamous cell carcinoma combined macrophage perturbation, promoter-occupancy and reporter assays to support CREB3L1-dependent SPP1 transcription within a CCR7–PERK signaling context [23]. That macrophage/tumor result cannot establish CREB3L1 regulation of *Spp1* in suture stromal cells. The present *Spp1* profiles do not measure protein export, matrix assembly or mineralization. The induction stains were separate culture-characterization assays and do not supply functional outcomes for the stretch or siRNA comparisons.

Matrix production may also feed back onto the physical stimulus. Imaging, modeling and perturbation experiments in embryonic mouse skull development identified feedback between tissue stiffness and cell fate that drives frontal-bone expansion [24]. This finding and the stiffness-dependent metabolic responses in mesenchymal cells [19] suggest that matrix production could modify the mechanical environment. Such changes could then alter stromal responses. A CREB3L1-associated collagen program might participate in such feedback, but its direction and effect cannot be predicted from *Col1a1* abundance alone. The distribution, organization and crosslinking of the deposited matrix would also matter. Our early transcript profiles and later Masson images neither establish this temporal sequence nor measure tissue stiffness. Developmental calvarial growth cannot be assumed to reproduce postnatal ZMS distraction.

A spatially restricted response is particularly relevant in a suture, where bone formation must coexist with an unmineralized interface. Bok et al. identified distinct CTSK-positive and DDR2-positive calvarial stem-cell lineages whose interactions influence mineralization and pathological fusion [1]. In zebrafish, Farmer et al. identified a mid-suture mesenchymal population expressing BMP antagonists, including *grem1a*, during suture establishment [25]. These studies suggest that osteogenic and restraining signals occupy distinct cellular contexts, rather than defining a uniform stromal response. Bone-marrow and periosteal progenitors likewise make different contributions to skeletal maintenance and repair [26]. Accordingly, overlap of *Creb3l1* and *Sp7* in our embedding could reflect a shared expression state without establishing cell identity, lineage conversion or direct regulation. The mixed expanded cultures cannot be assigned to a particular in vivo compartment from surface markers alone.

This compartmental view also changes the functional question. In *Fgfr2*-mutant Crouzon mice, cyclic cranial loading delayed coronal-suture and intersphenoidal-synchondrosis fusion and partly restored skull morphology [27]. This preclinical study links loading to preservation of growth interfaces without identifying a CREB3L1 mechanism. A recent triphasic scaffold study maintained a central stem-cell niche while promoting osteogenic differentiation in flanking regions [28]. These experiments show why a candidate regulator should ultimately be assessed against both matrix formation and preservation of sutural organization. For CREB3L1, the relevant outcome would be appropriately located matrix deposition with maintained suture function, rather than an increase in osteogenic transcripts across the entire interface. Neither our descriptive tissue time course nor the culture experiment establishes that spatial balance or supports a therapeutic claim.

Immune-derived signals offer an additional route by which loading could affect stromal matrix production. Related studies in this research program linked macrophage PDGF signaling to collagen remodeling and neutrophil-derived OSM to craniofacial bone regeneration [6,8]. These findings support considering paracrine inputs alongside direct stromal stimulation, but do not show that either ligand activates CREB3L1. The SPP1 and CXCL displays do not resolve this question. CellChat infers interactions from expression-compatible ligand–receptor relationships. Benchmarking demonstrates that predictions depend on both the interaction resource and inference method [9,29]. A focused test would determine whether a defined ligand or conditioned medium changes CREB3L1 cleavage and matrix output. It would then assess whether the response depends on stromal CREB3L1. The overlapping single-cell material provides contextual evidence rather than independent replication of the related publications.

The study has substantial limitations in biological replication, assay verification and reproducibility. Recent benchmarking of single-cell differential-expression methods emphasizes accounting for the nesting of cells within biological samples [30]; one sample per condition and time point cannot distinguish a distraction effect from sample variation. Archived count matrices are now supplied for independent reanalysis, but the original final-cell selection and panel-generating objects remain unavailable. Discrepancies between the documented integration/QC descriptions prevent verification of all settings behind the existing plots, so those displays cannot yet be reproduced exactly. Tissue qPCR groups are small, and exploratory exact testing does not correct limited power, incomplete *Osm* detection, high or discordant technical Cq values, reference-gene variation or unverified amplification efficiency. None of the 11 tissue comparisons was significant. This corrected non-rejection is structurally imposed by the combination of these sample sizes, exact-*p* discreteness and the 11-comparison family: even complete rank separation cannot meet the first Holm threshold. The analysis makes estimates and uncertainty transparent, but does not resolve inadequate biological replication or support absence of an effect. The four finite location-shift intervals included zero, while the other seven were unbounded, emphasizing limited precision under the stated model. No treatment-by-time interaction was tested. Culture qPCR has only one pooled donor preparation and one experimental run, despite separate vessel units; passage-specific identity remains unresolved. The four tissue IHC fields represent four author-confirmed mice, one per condition/day, without within-condition replication; precise animal/slide links remain unavailable. Culture-to-IF-field mappings are unresolved. Staining quantification and knockdown efficiency therefore cannot be reconstructed as replicated group outcomes. Micro-CT units and animal-level bilateral aggregation were clarified by the author, but numeric ROI dimensions, the exact HU threshold, original calibration measurements and specimen-linked acquisition settings remain unavailable. The expanded archived micro-CT series also lacks animal-level batch assignments, preventing adjustment for batch variation. Full flow-cytometry records, CREB3L1 cleavage measurements, rescue and matched functional matrix outcomes remain unavailable. Only male mice were studied. These limitations leave both reproducibility across biological samples and in vivo causality unresolved.

A focused next step would distinguish CREB3L1 abundance, processing and function under defined loading conditions [20,21]. Immunoblotting should resolve full-length CREB3L1 and its cleaved amino-terminal fragment, with validated controls; nuclear/cytoplasmic fractionation and compartment controls should test nuclear accumulation. Quantitative immunofluorescence with nuclear counterstaining and matched acquisition settings could provide a complementary nuclear-to-cytoplasmic measure. A CREB3L1-responsive reporter and measurements of targets such as *Col1a1* would test transcriptional activity, followed by newly synthesized/secreted collagen, ALP activity and matrix mineralization to assess function. Independently derived cultures should include static and stretched conditions with negative-control siRNA, verified CREB3L1 depletion and siRNA-resistant rescue, while monitoring viability and passage. Rescue with full-length protein versus its active amino-terminal fragment could distinguish a processing defect from downstream limitations. Current laboratory conditions preclude completing these additional experiments during this revision. When conditions permit, we plan follow-up studies with prospectively planned sample sizes, verified animal/donor identities, varied loading exposure and sampling times, and assay-specific passage records to test reproducibility and the proposed activation–function relationships. Native raw data, unit-level metadata, analysis code and computational objects will be retained for these future datasets; this prospective plan does not recover missing records from the present study. These are proposed experiments, not completed validation in the present study.

## 5. Conclusions

The study provides preliminary descriptions of CREB3L1-associated transcripts and staining in distracted mouse ZMS tissue and expanded suture-derived cultures. The small tissue comparisons were not statistically significant, and the culture observations do not establish equivalence or a reproducible absence of response. These observations guide preclinical studies of suture-directed craniofacial remodeling, where CREB3L1 processing, matrix function and preservation of suture patency require testing before therapeutic relevance can be established.

## Figures and Tables

**Table 1:** Study components, readouts and experimental units.

Study Component	Material and Conditions	Readouts	Experimental Units and Interpretation
Tissue morphology	SC/DO; days 1, 3, 7, 14 and 28	Micro-CT BV and regional BMD; H&E and Masson images	Micro-CT: 61 animals (31 SC/30 DO), bilateral means; endpoint counts and 10 exact tests in Table S5. Initial 46-animal account plus later same-condition animals; batch mapping unresolved. Histology qualitative.
Tissue expression	SC1/DO1/SC3/DO3; *Sp7*: SC7/DO7; IHC: days 1 and 3	*Spp1*, *Creb3l1* and *Sp7* qPCR; CREB3L1 IHC	*Spp1*/*Creb3l1*: 4 animals/group. *Sp7*: 2 SC7 and 5 DO7 animals. Exact tests with Holm correction across all 11 tissue comparisons; IHC: 1 animal/condition/day, 4 animals total; qualitative.
Single-cell context	SC1, DO1, SC3 and DO3; OMIX005296	Composition, stromal markers, trajectory and transcript displays	1 biological sample per condition/day; 4 libraries total. Existing overlapping dataset; no replicated SC–DO inference.
Culture characterization	ZMS explants from 6 donor mice; passages 3–7	Induction stains, 7 flow markers and culture morphology	Assay-specific vessel mapping and passage unresolved for characterization. Descriptive assays; no evidence of passage stability. Pooling was clarified specifically for the qPCR comparison.
Culture transcript profiles	Static (SC) and 3-day stretch (DO3), both with negative-control siRNA	*Creb3l1* and *Spp1* qPCR	2 SC dishes/3 DO3 chambers; one pooled preparation from 6 mice, one experiment. Mean ± SD across vessels; exploratory exact tests with Holm correction across 2 culture targets. No donor-level replication or equivalence test.
Culture immunostaining	Static negative-control/targeting siRNA; stretch with negative-control/targeting siRNA	CREB3L1 IF	Qualitative image sets; image/channel count is not biological *n*. No validated fluorescence-intensity or knockdown-efficiency estimate.
Extended tissue context	Same 4-library dataset; tissue qPCR in SC1/DO1/SC3/DO3	Enrichment, neutrophil subsets, SPP1/CXCL; *Osm*/*Lifr*/*Osmr* qPCR	*Lifr*/*Osmr*: 3 animals/group. *Osm*: 2/3/2/3 animals in SC1/DO1/SC3/DO3. Tests belong to the same 11-comparison family; computational displays descriptive.

SC denotes sham control in tissue and static negative-control-siRNA culture; DO denotes tissue distraction or in vitro stretch, as specified. Tissue qPCR points represent animals, and culture qPCR points represent separately cultured and treated dishes/chambers within one pooled cell preparation and one experiment. Technical wells are averaged within these units. Tissue tests are exploratory two-sided exact Mann–Whitney comparisons, with Holm correction across all 11 target-by-day comparisons. All qPCR plots show available points and mean ± SD on the log2 relative-expression scale. Culture SD describes between-vessel variation within that preparation, not between-donor or between-experiment variation. Single-cell displays contain one biological sample per condition and time point. Images are qualitative. The broader animal allocation is not an endpoint-specific sample size. ZMS, zygomaticomaxillary suture; qPCR, quantitative PCR; IHC, immunohistochemistry; IF, immunofluorescence; SD, standard deviation.

## Data Availability

The existing single-cell dataset is identified by OMIX accession OMIX005296 at https://ngdc.cncb.ac.cn/omix/release/OMIX005296 (accessed on 2 October 2026) [31]. This report retains existing visualizations. Four Single_Cell_Source_*.zip archives provide the recovered mouse count matrices, feature and barcode files, checksums and input-verification code for SC1, DO1, SC3 and DO3. These matrices and matched feature/barcode files permit independent reanalysis starting from the archived count-matrix inputs. However, the original 29,295-cell atlas subset and the displayed integration, annotation and trajectory panels cannot be reproduced because the final cell-selection metadata and panel-generating Seurat/Monocle2 objects remain unrecovered. Supplementary Data provides raw-well records from five selected qPCR instrument exports and all sample-level calculations for tissue *Spp1*, *Creb3l1*, *Sp7*, *Osm*, *Lifr* and *Osmr* and culture *Creb3l1* and *Spp1*. It includes source filenames and SHA256 hashes, worksheet/well identifiers, animal or culture-vessel codes, formulas, descriptive summaries, quality-control flags, the 11 exploratory tissue comparisons and the two exploratory culture-vessel comparisons, with separate Holm families. Tables S3 and S4 provide editable statistical summaries. The culture preparation and vessel mapping were clarified by the author during revision; they are not inferred from suffixes or contemporaneous instrument metadata. Original_qPCR_Exports.zip additionally supplies all eight original instrument exports, including measurements outside the selected analyses. Supplementary_Code.zip contains the executable tissue and culture verification scripts, run instructions and machine-readable statistical tables. Images and single-cell displays are supplied in the article and Supplementary Files. Original panel-generating single-cell objects and final cell-selection metadata, verified independent-unit staining quantification, targeting-siRNA raw Cq measurements, and CREB3L1 cleavage/rescue data are not included in the available study materials. The single-cell displays and qualitative staining results cannot be fully reproduced or independently quantified from this reporting package. MicroCT_Source_Data.zip supplies the original micro-CT workbook, all 66 ordinary ZMS-SC/DO source rows, the 61 reconstructed animal-level bilateral means, explicit exclusions and missingness, author-clarified metadata, executable analysis code, Table S5 data and checksums. The archive reproduces calculations from recorded values; it does not reproduce image segmentation or recover the original calibration curve or batch assignments.

## Supplementary Materials

The following supporting information can be downloaded at: https://www.mdpi.com/article/10.3390/biology15211918/s1, Figure S1: Qualitative suture histology across the sampling period; Figure S2: Qualitative CREB3L1 immunofluorescence in expanded suture-derived cultures; Figure S3: Enrichment and protein-association displays for neutrophils; Figure S4: Enrichment and protein-association displays for monocytes; Figure S5: Enrichment and protein-association displays for fibroblast-like stromal cells; Figure S6: Extended neutrophil transcriptional displays; Figure S7: Predicted pathway and interaction displays involving SPP1; Figure S8: OSM-related expression, tissue qPCR and CXCL predictions; Tables S1–S5: Single-cell methods, primers and exploratory statistics; Supplementary Data: Source data, calculations and exploratory qPCR statistics; Supplementary Code: Reproducible qPCR analyses; Supplementary Single-Cell Source Data: Archived mouse count-matrix inputs; Supplementary Original qPCR Exports: Complete archived instrument files; Supplementary Micro-CT Source Data: Archived measurements and reanalysis.

## Abbreviations

CREB3L1, cAMP responsive element binding protein 3 like 1; CT, computed tomography; DAB, 3,3′-diaminobenzidine; DAPI, 4′,6-diamidino-2-phenylindole; DMEM, Dulbecco’s modified Eagle’s medium; DO, distraction in vivo or cyclic stretch in vitro, as specified; EDTA, ethylenediaminetetraacetic acid; FCS, flow cytometry standard; GO, Gene Ontology; H&E, hematoxylin and eosin; IF, immunofluorescence; IHC, immunohistochemistry; KEGG, Kyoto Encyclopedia of Genes and Genomes; MPCs, legacy cell-population label retained without a lineage assignment; OPN, osteopontin (protein encoded by *Spp1*); OSM, oncostatin M; PPI, protein–protein interaction; qPCR, quantitative polymerase chain reaction; SC, sham control in vivo or static culture in vitro, as specified; SCT, SCTransform; SDSCs, suture-derived stromal cells; SD, standard deviation; siRNA, small interfering RNA; UMAP, uniform manifold approximation and projection; ZMS, zygomaticomaxillary suture.
