# Supplementary material for: CREB3L1-Associated Stromal Expression During Mechanical Remodeling of the Mouse Zygomaticomaxillary Suture
Source: Biology (Basel). 2026 Oct 23;15(21):1918. doi: 10.3390/biology15211918 (PMC13762063; doi:10.3390/biology15211918)
Supplement: Supplementary file 1 [file biology-15-01918-s001.zip › Supplementary_Tables.pdf]

Supplementary Materials

Supplementary Tables S1–S5

CREB3L1-associated stromal expression during mechanical remodeling of the mouse zygomaticomaxillary suture

Table S1 Single-cell methods and reproducibility

Stage	Recorded procedure	Verification and interpretation
Libraries	SC1, DO1, SC3, DO3; 29,295 cells; OMIX005296; SeekOne MM 3′ and NovaSeq 6000	One biological sample per condition/day; overlapping dataset. Four mouse input matrices (43,405 barcodes) are supplied separately. Original 29,295-cell selection and panel-generating objects remain unavailable; displayed analyses were not rerun.
Atlas integration and PCA	Seurat 4.3.0 / R 4.2.2. Narrative: CCA → PCA → UMAP. Appendix: SCT integration, 3,000 features, 30 PCs, resolution 0.25.	PCA is documented. Integration and QC descriptions differ. Final execution and every display-generating parameter cannot be verified.
Stromal subclustering	Appendix: LogNormalize (10,000), 2,000 variable features, 20 PCs; neighbors 1–20, resolution 0.1, UMAP 1–10.	Recorded appendix settings; final objects unavailable. *Spp1*-Fib/*Ptprb*-Fib are descriptive expression annotations, not validated lineages.
Trajectory	Monocle2 2.26.0; DDRTree embedding and relative cell ordering.	Relative pseudotime and computational state do not establish elapsed time, lineage transition or CREB3L1 activity.
Communication	CellChat 1.6.1; CellChatDB.mouse; secreted signaling, PPI.mouse and minimum 10 cells; pathway probabilities and aggregation.	Expression-based predictions. Final execution not reproduced. MPCs remains an undefined source label.
Enrichment	Existing GO/KEGG and protein-association displays. Figure S5b is GO molecular function.	Full gene lists, backgrounds and final output objects unavailable; calculations not rerun. No replicated animal-level distraction inference.

The narrative and dissertation appendix differ in integration and filtering settings. Archived displays were not rerun; final panel-generating parameters remain unverified. SCT, SCTransform; PCA, principal component analysis; UMAP, uniform manifold approximation and projection.

Table S2 Quantitative PCR primers

Gene	Forward primer 5′ to 3′	Reverse primer 5′ to 3′
Creb3l1	GCCCTGGGAAACAAGCTGT	AGCTGAGTCATTTCTCCTGGG
Sp7	ATGGCGTCCTCTCTGCTTG	TGAAAGGTCAGCGTATGGCTT
Spp1	AGCAAGAAACTCTTCCAAGCAA	GTGAGATTCGTCAGATTCATCCG
Osm	CCCGGCACAATATCCTCGG	TCTGGTGTTGTAGTGGACCGT
Lifr	AGCTCTGACCCTCCTGCAT	TGGGTGACAAGAATGGAACCT
Osmr	CATCCCGAAGCGAAGTCTTGG	GGCTGGGACAGTCCATTCTAAA
Gapdh	AGGTCGGTGTGAACGGATTTG	TGTAGACCATGTAGTTGAGGTCA

Forward and reverse primer sequences are written 5′ to 3′ and were transcribed from the thesis and original manuscript materials. *Gapdh* was the reference gene. Nomenclature follows Section 2.7 of the manuscript: mouse gene/transcript symbols are italic (e.g., *Creb3l1* and *Spp1*), and protein names are roman (CREB3L1 and osteopontin, OPN). Original assay labels "CREB" and "Opn"/"OPN" map to *Creb3l1* and *Spp1*, respectively, and remain preserved in raw source records and provenance fields. CellChat pathway names (e.g., SPP1) and protein-association network identifiers remain roman database labels. Primer transcription does not establish amplification efficiency or assay specificity. Sample-level Cq records, animal or culture-vessel identities, calculations and quality-control flags for the qPCR plots are provided in Supplementary Data.

Table S3 Exploratory exact comparisons of tissue qPCR expression

A Descriptive animal-level effects. Positive DO−SC differences indicate higher distraction-group expression. SC means are zero by same-day calibration. SD describes dispersion among available animals.

Target	Day	SC n	DO n	SC mean ± SD	DO mean ± SD	Mean difference log2 DO−SC	Geometric mean ratio DO/SC
Creb3l1	1	4	4	0.000 ± 0.648	-1.299 ± 0.640	-1.299	0.406
Creb3l1	3	4	4	0.000 ± 1.176	1.853 ± 1.285	+1.853	3.612
Spp1	1	4	4	0.000 ± 0.980	-1.420 ± 1.507	-1.420	0.374
Spp1	3	4	4	0.000 ± 1.353	1.191 ± 0.643	+1.191	2.283
Sp7	7	2	5	0.000 ± 0.935	1.045 ± 1.624	+1.045	2.063
Osm	1	2	3	0.000 ± 0.452	1.850 ± 0.939	+1.850	3.604
Osm	3	2	3	0.000 ± 1.146	2.269 ± 0.636	+2.269	4.819
Lifr	1	3	3	0.000 ± 0.630	-0.933 ± 0.896	-0.933	0.524
Lifr	3	3	3	0.000 ± 0.456	1.445 ± 1.052	+1.445	2.723
Osmr	1	3	3	0.000 ± 0.546	0.150 ± 0.500	+0.150	1.110
Osmr	3	3	3	0.000 ± 0.718	1.045 ± 0.618	+1.045	2.064

The ratio of geometric means equals 2 raised to the mean log2 difference. It is a descriptive effect; the rank-based intervals on the following page apply to a separate location-shift parameter. Technical wells were averaged within each animal. Missing values were not imputed; all finite values, including flagged Cq measurements, were retained.

Table S3 Exploratory exact comparisons of tissue qPCR expression continued

B Exact inference and location-shift precision. All 11 comparisons form one Holm family. HL denotes the Hodges–Lehmann median of cross-group DO−SC differences.

Target	Day	U DO	Rank-biserial DO−SC	Exact P	Holm P all 11	HL shift log2	HL interval log2	Achieved coverage
Creb3l1	1	1	-0.875	0.0571	0.6286	-1.732	[-2.268, 0.595]	97.14%
Creb3l1	3	14	+0.750	0.1143	1.0000	+1.856	[-0.534, 5.000]	97.14%
Spp1	1	3	-0.625	0.2000	1.0000	-1.558	[-3.537, 1.729]	97.14%
Spp1	3	13	+0.625	0.2000	1.0000	+1.303	[-0.811, 3.704]	97.14%
Sp7	7	7	+0.400	0.5714	1.0000	+0.659	(−∞, +∞)	100%
Osm	1	6	+1.000	0.2000	1.0000	+1.780	(−∞, +∞)	100%
Osm	3	6	+1.000	0.2000	1.0000	+2.106	(−∞, +∞)	100%
Lifr	1	1	-0.778	0.2000	1.0000	-0.932	(−∞, +∞)	100%
Lifr	3	9	+1.000	0.1000	1.0000	+1.295	(−∞, +∞)	100%
Osmr	1	7	+0.556	0.4000	1.0000	+0.126	(−∞, +∞)	100%
Osmr	3	9	+1.000	0.1000	1.0000	+1.220	(−∞, +∞)	100%

Raw P values are two-sided exact Mann–Whitney values with U referring to DO; positive rank-biserial values indicate higher DO ranks. HL intervals are marginal equal-tailed exact intervals with at least 95% coverage, assuming independent continuous distributions of common shape differing by a location shift. They are not multiplicity-adjusted and are not confidence intervals for the mean difference or geometric-mean ratio. Discreteness gives 97.142857% coverage for the four finite intervals. The remaining seven 95% intervals are unbounded, reflecting insufficient precision for finite equal-tailed exact bounds.

Observed raw P values all exceed 0.05. With these group sizes, the minimum attainable raw exact P of 0.0286 exceeds the first Holm threshold of 0.00455, making adjusted significance unattainable even with complete rank separation. Nonsignificance does not establish equivalence or absence of a response. Amplification efficiency, *Gapdh* stability, NTC and melt-curve performance remain unverified. Culture-vessel comparisons are reported separately in Table S4; all five vessels came from one pooled donor preparation and one experiment. Raw records, formulas, QC flags and the reproducible exact-analysis script accompany the revised source workbook.

Supplementary Micro-CT Source Data. Archived measurements and reanalysis

File: MicroCT_Source_Data.zip

The archive contains the original workbook, ordinary ZMS-SC/DO source records, 61 animal-level bilateral means, author-clarified units and aggregation rules, analysis code, Table S5 data and checksums. Four blank records remain missing; GANT and CS components are excluded. The code reproduces calculations from recorded values, including the DO1-3 bilateral average, exact tests with ties, a separate 10-test Holm correction and marginal shift intervals. Original segmentation, phantom calibration and batch assignments are not recovered. No new animals or experiments were added during this revision.

Table S4 Exploratory culture-vessel qPCR comparisons

Two static dishes and three separately treated stretch chambers were studied in one experiment using one cell preparation pooled from six donor mice. Each vessel supplied one RNA sample; two qPCR wells per target were averaged within that sample. The vessels are the units in this table. They are not independent donor preparations or repeated experiments.

Target	SC vessels	DO3 vessels	SC mean ± SD	DO3 mean ± SD	Mean DO3−SC	Geometric-mean ratio	U (DO3)	Exact P	Holm P
Creb3l1	2	3	0.000 ± 0.013	−0.092 ± 0.373	−0.092	0.938	2	0.800	1.000
Spp1	2	3	0.000 ± 0.797	0.105 ± 0.447	0.105	1.076	3	1.000	1.000

Means and SDs are on the log2 relative-expression scale. SC means are zero by calibration to the mean SC ΔCq; control variation is retained. The geometric-mean ratio equals 2 raised to the descriptive mean log2 difference. Culture SD reflects vessel variation within this pooled preparation and experiment.

P values are two-sided exact Mann–Whitney values from all 10 allocations of two SC labels among five vessels. The two culture targets form a separate Holm family from the 11 tissue comparisons in Table S3. These exploratory tests assume independent and exchangeable vessel-level measurements under the null; random allocation was not documented. They do not quantify between-donor or between-experiment reproducibility or establish a causal treatment effect.

The Hodges–Lehmann location shifts (DO3−SC) are −0.238 for *Creb3l1* and +0.064 for *Spp1*. Equal-tailed exact intervals at the 95% level are unbounded (−∞ to +∞) for both under a continuous common-shape shift model; they are not intervals for the mean differences or geometric-mean ratios. The minimum attainable two-sided exact P at n = 2 versus 3 is 0.200. Nonsignificance cannot establish equivalence or absence of a response. No matched-pair analysis was performed.

Vessel identity, separate handling, pooling and the single-run design were clarified by the author during revision. The source instrument records and all numeric values are unchanged. Raw wells, culture-vessel calculations, QC flags and reproducible code accompany the source workbook.

Table S5 Archived micro-CT measurements: animal-level estimates

BV is bone volume (mm³); regional BMD is hydroxyapatite-equivalent bone mineral density over the whole ROI (mg HA/cm³). Each animal contributes one bilateral mean. Different animals were sampled at each day. Mean differences and ratios below are descriptive, not the parameters tested by the rank test.

Endpoint	Day	SC n	DO n	SC mean ± SD	DO mean ± SD	Mean DO−SC	Mean ratio DO/SC
BV	1	6	6	0.708 ± 0.042	0.764 ± 0.056	0.056	1.079
BV	3	7	5	0.710 ± 0.054	0.782 ± 0.064	0.072	1.101
BV	7	6	7	0.853 ± 0.082	0.937 ± 0.099	0.084	1.098
BV	14	6	6	0.858 ± 0.101	1.212 ± 0.133	0.353	1.412
BV	28	6	6	0.755 ± 0.043	0.950 ± 0.137	0.195	1.258
Regional BMD	1	6	6	1687.765 ± 30.592	1664.217 ± 30.660	−23.547	0.986
Regional BMD	3	7	5	1746.312 ± 20.066	1681.631 ± 18.101	−64.681	0.963
Regional BMD	7	6	7	1723.678 ± 28.435	1729.684 ± 31.676	6.005	1.003
Regional BMD	14	6	6	1746.039 ± 20.938	1729.004 ± 20.591	−17.035	0.990
Regional BMD	28	6	6	1871.748 ± 6.620	1804.009 ± 30.111	−67.739	0.964

The ordinary ZMS-SC/DO subset contains 66 labelled rows: four missing numerical records and 62 valid measurements. The two ZMS-DO1-3 rows are left/right regions of one mouse and are averaged; all other single entries already represent bilateral means. The resulting 61 animals comprise 31 SC and 30 DO mice. GANT and CS components are outside this comparison; missing values were not imputed.

The original 46-animal account covers only part of the study. The author confirmed that this archive additionally includes animals studied later under the same conditions; it is not a new experiment or independent replication. Individual batch identities and overlap with other endpoints remain unresolved. All source values, the author-clarified metadata and aggregation rules are in MicroCT_Source_Data.zip.

Table S5 (continued) Exact tests and marginal location-shift intervals

Endpoint	Day	U (DO)	Exact P	Holm P	HL shift DO−SC	Marginal 95% shift interval
BV	1	29.5	0.07143	0.42857	0.055	−0.020 to 0.115
BV	3	28.0	0.08838	0.44192	0.070	0.000 to 0.160
BV	7	32.5	0.10897	0.44192	0.085	−0.050 to 0.210
BV	14	35.0	0.00433	0.03463	0.360	0.200 to 0.500
BV	28	33.5	0.01082	0.07576	0.180	0.040 to 0.350
Regional BMD	1	7.0	0.09307	0.44192	−20.661	−58.768 to 6.888
Regional BMD	3	0.0	0.00253	0.02273	−62.408	−90.991 to −42.263
Regional BMD	7	23.0	0.83566	0.83566	2.287	−32.387 to 45.244
Regional BMD	14	11.0	0.30952	0.61905	−18.091	−44.339 to 16.038
Regional BMD	28	0.0	0.00216	0.02165	−69.118	−98.398 to −24.297

All group-label allocations were enumerated. Two-sided P values compare the absolute distance of the observed U statistic from its null center, retaining ties. All 10 micro-CT tests (two endpoints at five time points) form one Holm family, separate from the 11 tissue qPCR tests and two culture-vessel tests. No comparisons were dropped: this table reports all 10 raw and adjusted P values, including the seven adjusted values that were not significant. Exploratory family-wise significance uses adjusted $P < 0.05$. No treatment-by-time interaction or batch adjustment was performed.

HL is the Hodges–Lehmann estimate, the median of all DO−SC pairwise differences. Intervals invert the same tie-aware permutation test under an independent, common-shape additive-shift model; all 10 accepted shift sets were connected. These are unadjusted marginal 95% location-shift intervals, not intervals for mean differences or ratios. The full inversion and permutation distributions are supplied with executable code.

The author clarified fixed-size ZMS-centered ROIs, a common bone threshold, HU units and phantom-based conversion $BMD = (\text{mean HU} + 739.11)/1.3487$. Exact ROI dimensions, the numeric HU threshold, original phantom measurements and specimen-linked scan parameters remain unavailable. Consequently, recorded-value calculations can be reproduced but original segmentation/calibration and batch effects cannot be independently verified.
