# Supplementary material for: CREB3L1-Associated Stromal Expression During Mechanical Remodeling of the Mouse Zygomaticomaxillary Suture
Source: Biology (Basel). 2026 Oct 23;15(21):1918. doi: 10.3390/biology15211918 (PMC13762063; doi:10.3390/biology15211918)
Supplement: Supplementary file 1 [file biology-15-01918-s001.zip › Supplementary_Figures.zip!/Supplementary_Figures/Figure_S8.pdf]

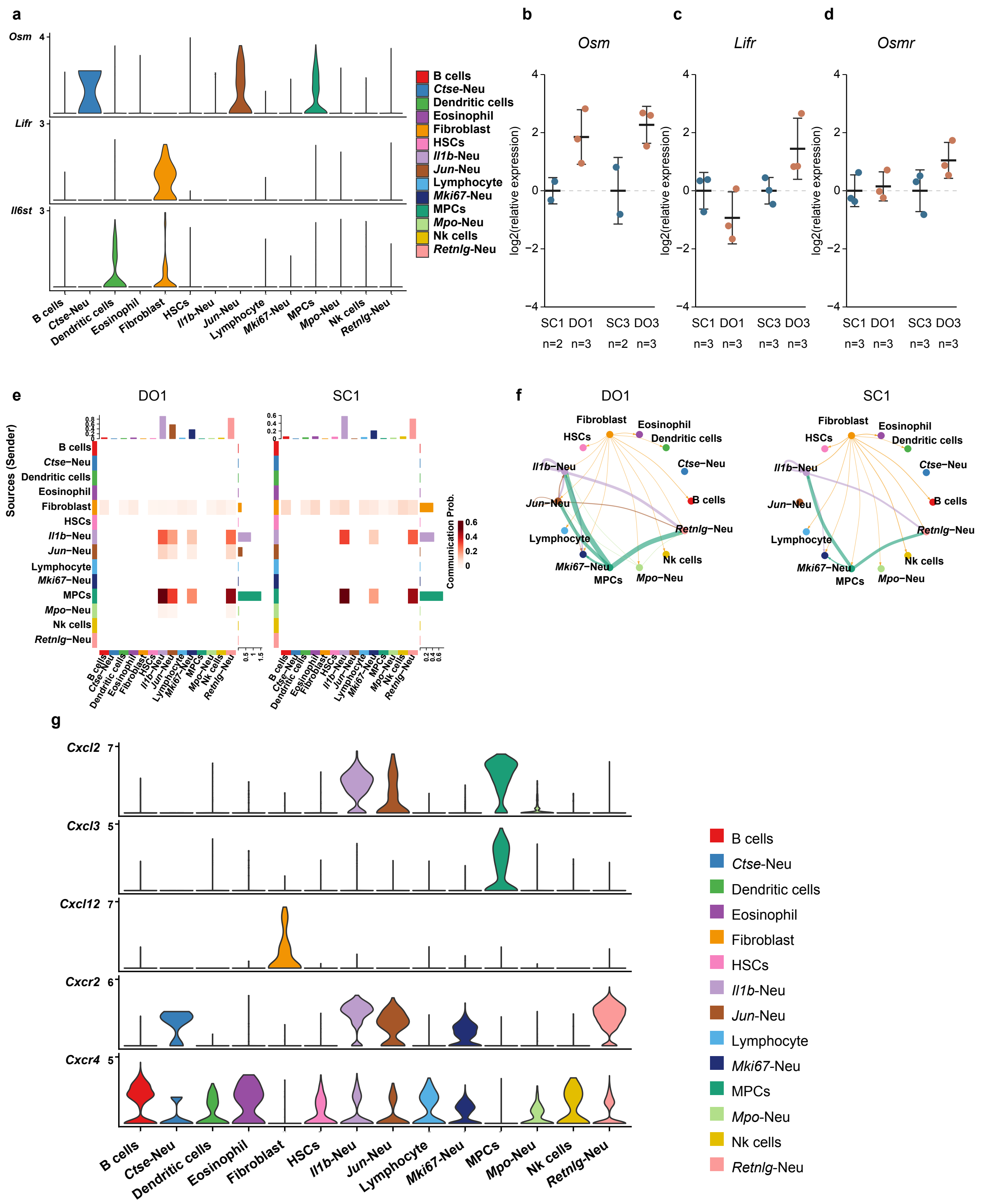

a
Osm
Lifr
Il6st
B cells
Ctse-Neu
Dendritic cells
Eosinophil
Fibroblast
HSCs
Il1b-Neu
Jun-Neu
Lymphocyte
Mki67-Neu
MPCs
Mpo-Neu
Nk cells
Retnlg-Neu
b
Osm
c
Lifr
d
Osmr
log2(relative expression)
SC1 DO1 SC3 DO3
n=2 n=3 n=2 n=3
n=3 n=3 n=3 n=3
n=3 n=3 n=3 n=3
e
DO1
SC1
Sources (Sender)
Communication Prob.
f
DO1
SC1
g
Cxcl2
Cxcl3
Cxcl12
Cxcr2
Cxcr4
