# Supplementary material for: CREB3L1-Associated Stromal Expression During Mechanical Remodeling of the Mouse Zygomaticomaxillary Suture
Source: Biology (Basel). 2026 Oct 23;15(21):1918. doi: 10.3390/biology15211918 (PMC13762063; doi:10.3390/biology15211918)
Supplement: Supplementary file 1 [file biology-15-01918-s001.zip › Supplementary_Figures.zip!/Supplementary_Figures/Figure_S3.pdf]

a

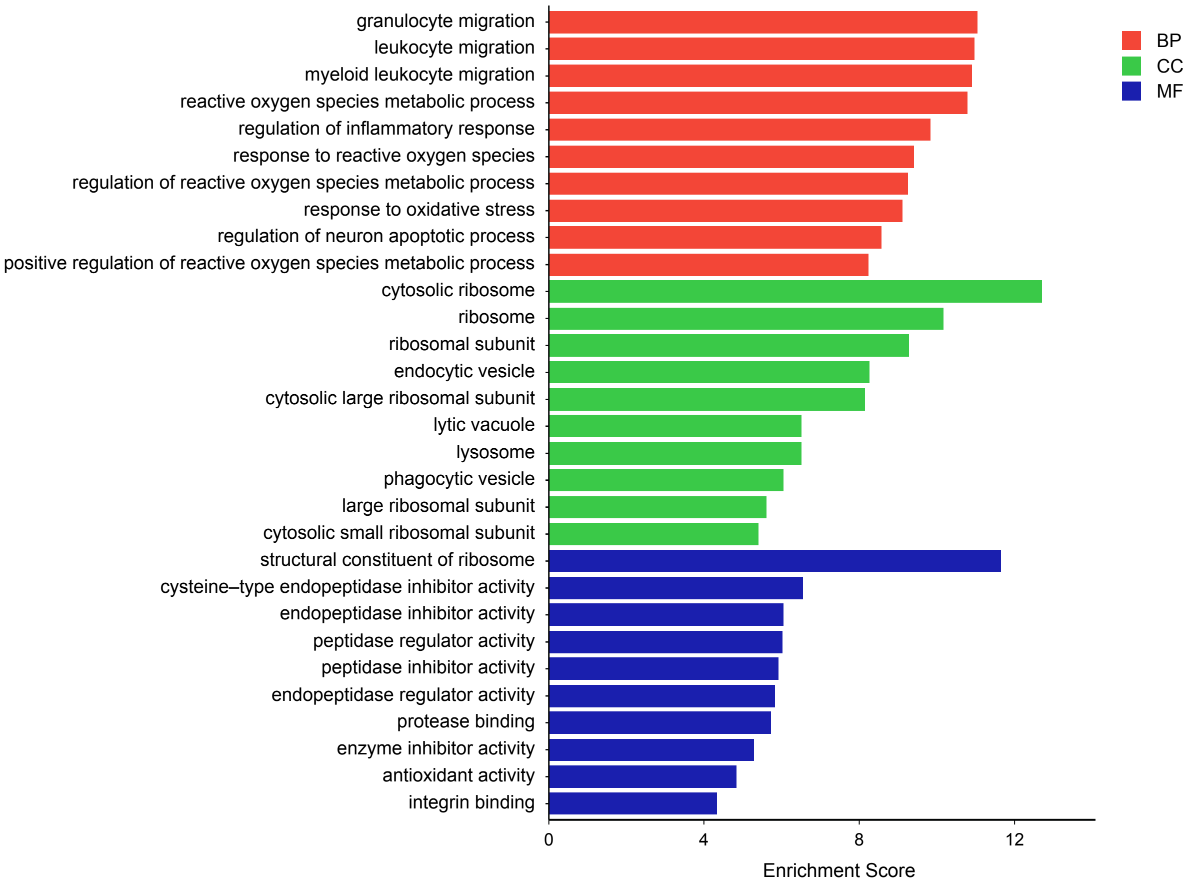
granulocyte migration
leukocyte migration
myeloid leukocyte migration
reactive oxygen species metabolic process
regulation of inflammatory response
response to reactive oxygen species
regulation of reactive oxygen species metabolic process
response to oxidative stress
regulation of neuron apoptotic process
positive regulation of reactive oxygen species metabolic process
cytosolic ribosome
ribosome
ribosomal subunit
endocytic vesicle
cytosolic large ribosomal subunit
lytic vacuole
lysosome
phagocytic vesicle
large ribosomal subunit
cytosolic small ribosomal subunit
structural constituent of ribosome
cysteine–type endopeptidase inhibitor activity
endopeptidase inhibitor activity
peptidase regulator activity
peptidase inhibitor activity
endopeptidase regulator activity
protease binding
enzyme inhibitor activity
antioxidant activity
integrin binding
0
4
8
12
Enrichment Score
BP
CC
MF

b

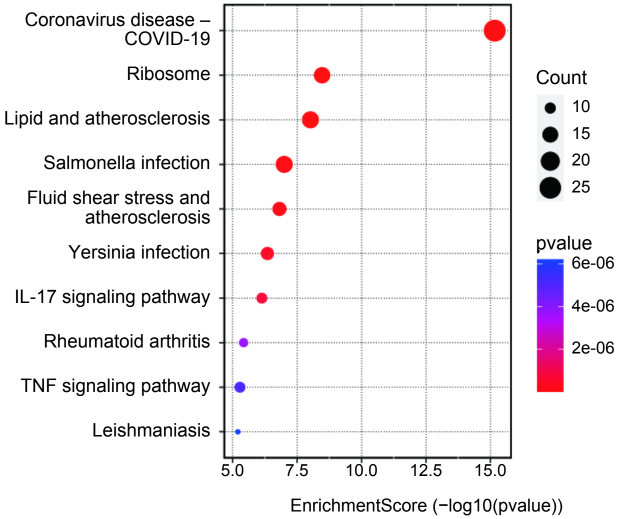
Coronavirus disease – COVID-19
Ribosome
Lipid and atherosclerosis
Salmonella infection
Fluid shear stress and atherosclerosis
Yersinia infection
IL-17 signaling pathway
Rheumatoid arthritis
TNF signaling pathway
Leishmaniasis
5.0
7.5
10.0
12.5
15.0
EnrichmentScore (−log10(pvalue))
Count
10
15
20
25
pvalue
6e-06
4e-06
2e-06

c

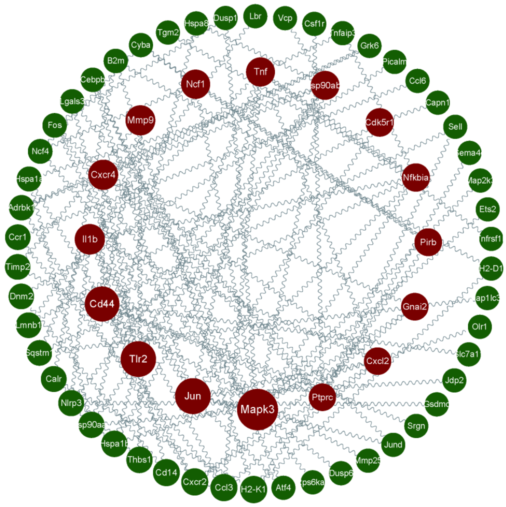
Tnf
Ncf1
Mmp9
Cdk5r1
Cxcr4
Nfkbia
Il1b
Pirb
Cd44
Gnai2
Tlr2
Cxcl2
Jun
Mapk3
Ptprc
Lbr
Vcp
Dusp1
Csf1r
Grk6
Cyba
Tgm2
B2m
Ccl6
Fos
Sell
Ncf4
Ccr1
Timp2
Dnm2
Ets2
Olr1
Calr
Nlrp3
Jdp2
Srgn
Jund
Thbs1
Cd14
Cxcr2
Ccl3
H2-K1
Atf4
Dusp6
