# Supplementary material for: CREB3L1-Associated Stromal Expression During Mechanical Remodeling of the Mouse Zygomaticomaxillary Suture
Source: Biology (Basel). 2026 Oct 23;15(21):1918. doi: 10.3390/biology15211918 (PMC13762063; doi:10.3390/biology15211918)
Supplement: Supplementary file 1 [file biology-15-01918-s001.zip › Supplementary_Figures.zip!/Supplementary_Figures/Figure_S2.pdf]

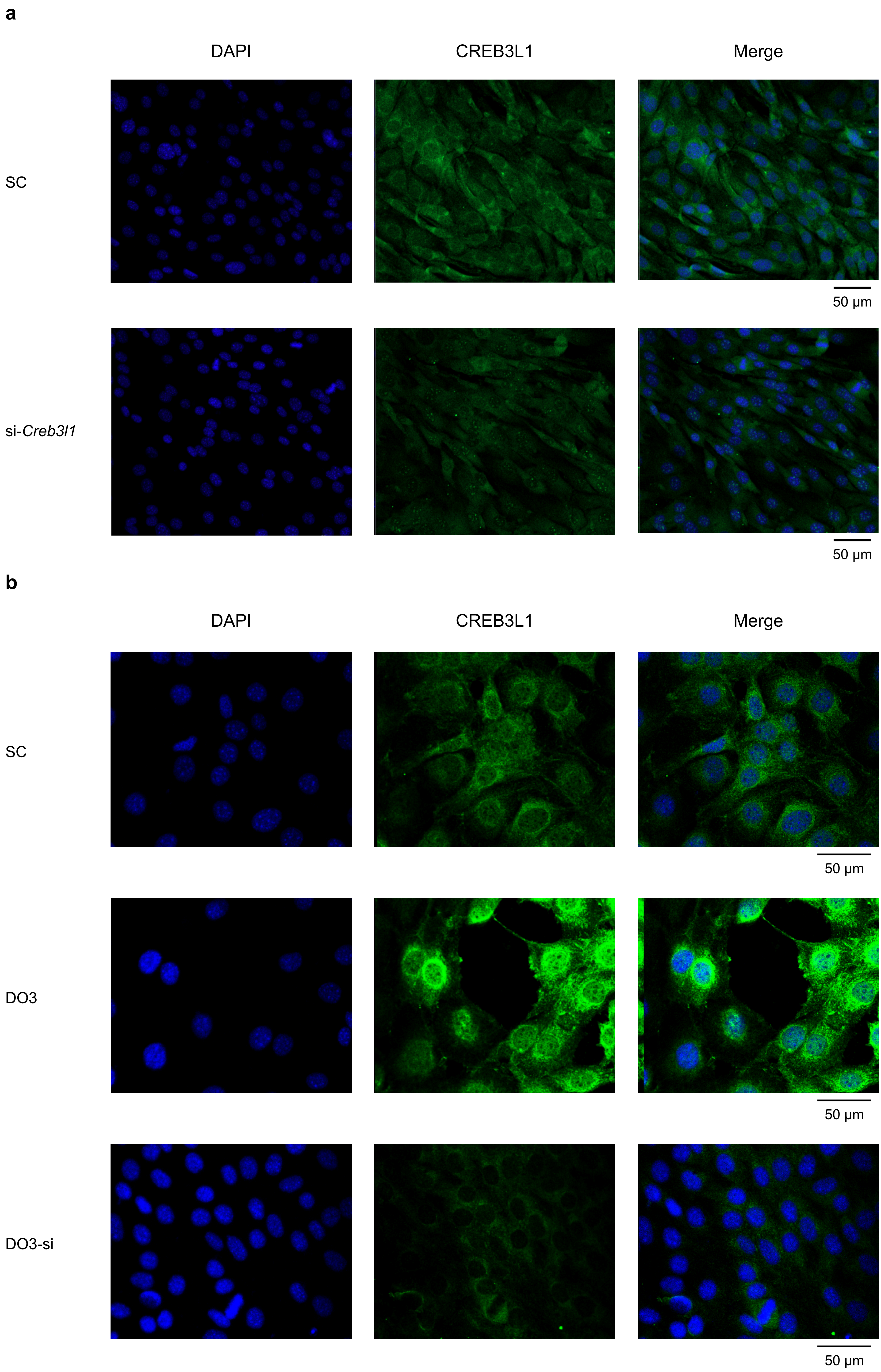
a
DAPI
CREB3L1
Merge
SC
50 μm
si-*Creb3l1*
50 μm
b
DAPI
CREB3L1
Merge
SC
50 μm
DO3
50 μm
DO3-si
50 μm
