# Supplementary material for: CREB3L1-Associated Stromal Expression During Mechanical Remodeling of the Mouse Zygomaticomaxillary Suture
Source: Biology (Basel). 2026 Oct 23;15(21):1918. doi: 10.3390/biology15211918 (PMC13762063; doi:10.3390/biology15211918)
Supplement: Supplementary file 1 [file biology-15-01918-s001.zip › Supplementary_Figures.zip!/Supplementary_Figures/Figure_S1.pdf]

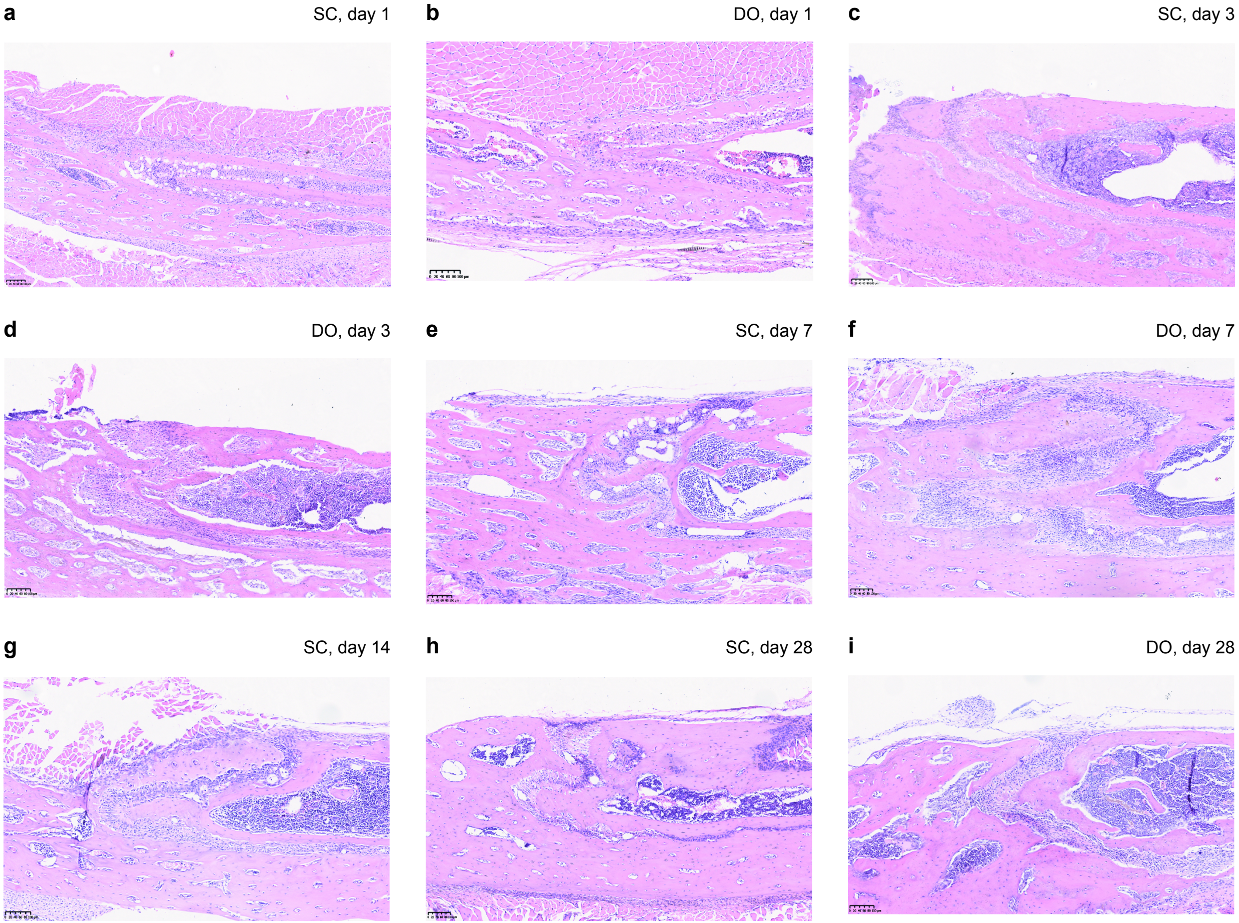
a
SC, day 1
b
DO, day 1
c
SC, day 3
d
DO, day 3
e
SC, day 7
f
DO, day 7
g
SC, day 14
h
SC, day 28
i
DO, day 28
