# Supplementary material for: CREB3L1-Associated Stromal Expression During Mechanical Remodeling of the Mouse Zygomaticomaxillary Suture
Source: Biology (Basel). 2026 Oct 23;15(21):1918. doi: 10.3390/biology15211918 (PMC13762063; doi:10.3390/biology15211918)
Supplement: Supplementary file 1 [file biology-15-01918-s001.zip › Supplementary_Figures.zip!/Supplementary_Figures/Figure_S5.pdf]

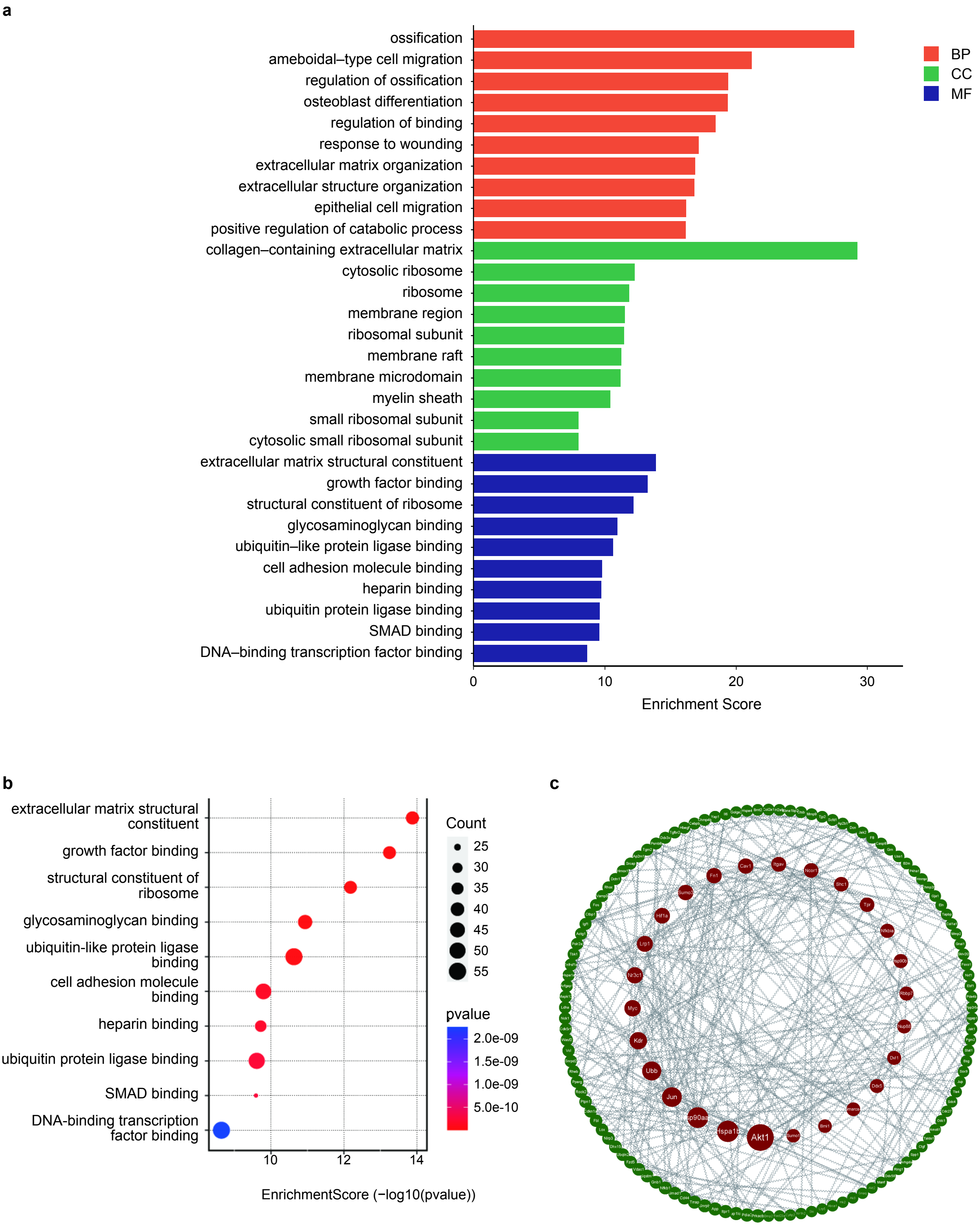
a
ossification
ameboidal–type cell migration
regulation of ossification
osteoblast differentiation
regulation of binding
response to wounding
extracellular matrix organization
extracellular structure organization
epithelial cell migration
positive regulation of catabolic process
collagen–containing extracellular matrix
cytosolic ribosome
ribosome
membrane region
ribosomal subunit
membrane raft
membrane microdomain
myelin sheath
small ribosomal subunit
cytosolic small ribosomal subunit
extracellular matrix structural constituent
growth factor binding
structural constituent of ribosome
glycosaminoglycan binding
ubiquitin–like protein ligase binding
cell adhesion molecule binding
heparin binding
ubiquitin protein ligase binding
SMAD binding
DNA–binding transcription factor binding
BP
CC
MF
0
10
20
30
Enrichment Score
b
extracellular matrix structural constituent
growth factor binding
structural constituent of ribosome
glycosaminoglycan binding
ubiquitin-like protein ligase binding
cell adhesion molecule binding
heparin binding
ubiquitin protein ligase binding
SMAD binding
DNA-binding transcription factor binding
10
12
14
EnrichmentScore (−log10(pvalue))
Count
25
30
35
40
45
50
55
pvalue
2.0e-09
1.5e-09
1.0e-09
5.0e-10
c
Akt1
Hspa1b
Jun
Ubb
Kdr
Myc
Lrp1
Fn1
Cav1
Itgav
Ncor1
Shc1
Tpr
