# Supplementary material for: CREB3L1-Associated Stromal Expression During Mechanical Remodeling of the Mouse Zygomaticomaxillary Suture
Source: Biology (Basel). 2026 Oct 23;15(21):1918. doi: 10.3390/biology15211918 (PMC13762063; doi:10.3390/biology15211918)
Supplement: Supplementary file 1 [file biology-15-01918-s001.zip › Supplementary_Figures.zip!/Supplementary_Figures/Figure_S4.pdf]

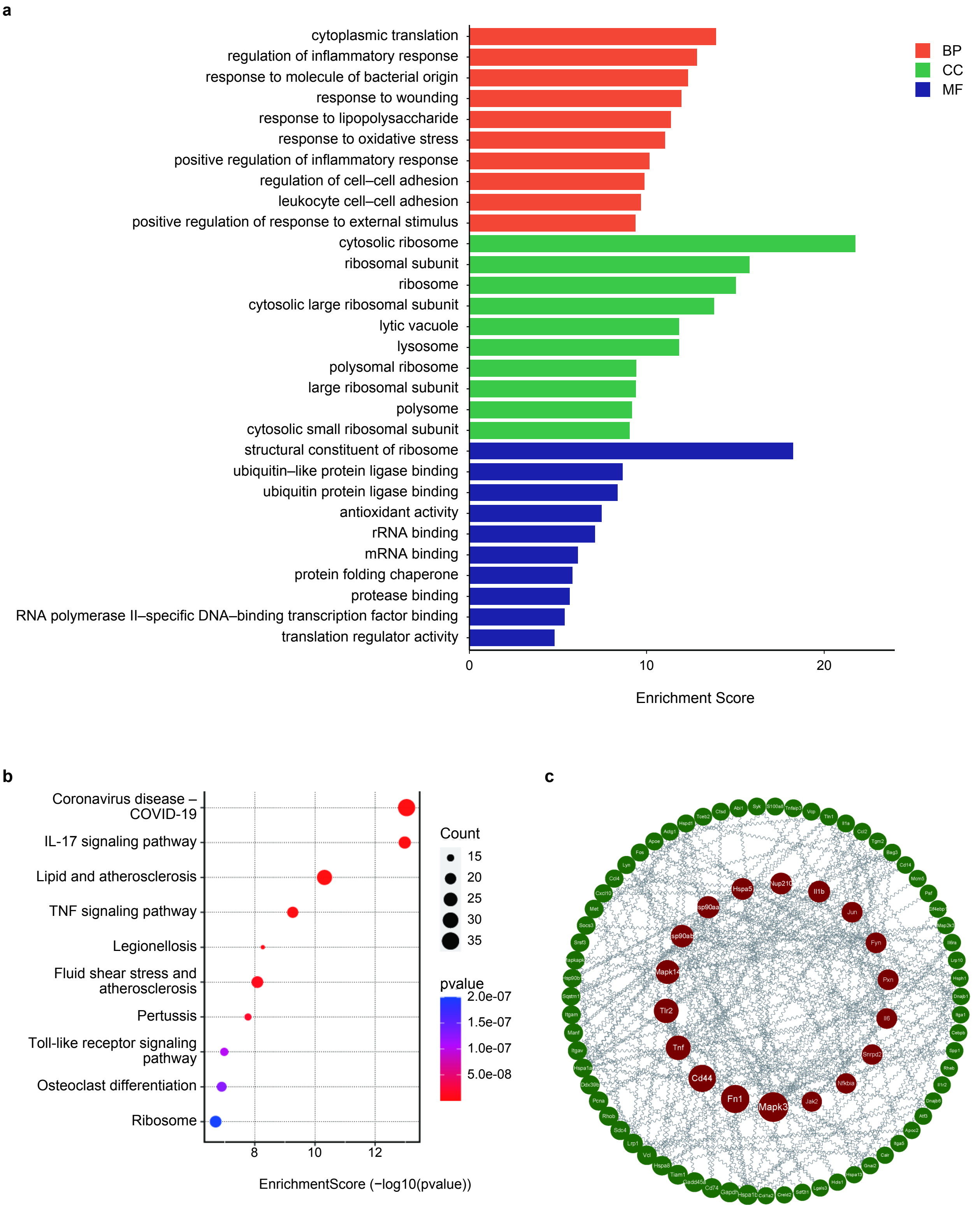
a
cytoplasmic translation
regulation of inflammatory response
response to molecule of bacterial origin
response to wounding
response to lipopolysaccharide
response to oxidative stress
positive regulation of inflammatory response
regulation of cell–cell adhesion
leukocyte cell–cell adhesion
positive regulation of response to external stimulus
cytosolic ribosome
ribosomal subunit
ribosome
cytosolic large ribosomal subunit
lytic vacuole
lysosome
polysomal ribosome
large ribosomal subunit
polysome
cytosolic small ribosomal subunit
structural constituent of ribosome
ubiquitin–like protein ligase binding
ubiquitin protein ligase binding
antioxidant activity
rRNA binding
mRNA binding
protein folding chaperone
protease binding
RNA polymerase II–specific DNA–binding transcription factor binding
translation regulator activity
0
10
20
Enrichment Score
BP
CC
MF
b
Coronavirus disease – COVID-19
IL-17 signaling pathway
Lipid and atherosclerosis
TNF signaling pathway
Legionellosis
Fluid shear stress and atherosclerosis
Pertussis
Toll-like receptor signaling pathway
Osteoclast differentiation
Ribosome
8
10
12
EnrichmentScore (−log10(pvalue))
Count
15
20
25
30
35
pvalue
2.0e-07
1.5e-07
1.0e-07
5.0e-08
c
Hspa5
Nup210
Il1b
sp90aa
Jun
sp90ab
Fyn
Mapk14
Pxn
Tlr2
Il6
Tnf
Snrpd2
Cd44
Nfkbia
Fn1
Jak2
Mapk3
