# Supplementary material for: CREB3L1-Associated Stromal Expression During Mechanical Remodeling of the Mouse Zygomaticomaxillary Suture
Source: Biology (Basel). 2026 Oct 23;15(21):1918. doi: 10.3390/biology15211918 (PMC13762063; doi:10.3390/biology15211918)
Supplement: Supplementary file 1 [file biology-15-01918-s001.zip › Supplementary_Figures.zip!/Supplementary_Figures/Figure_S6.pdf]

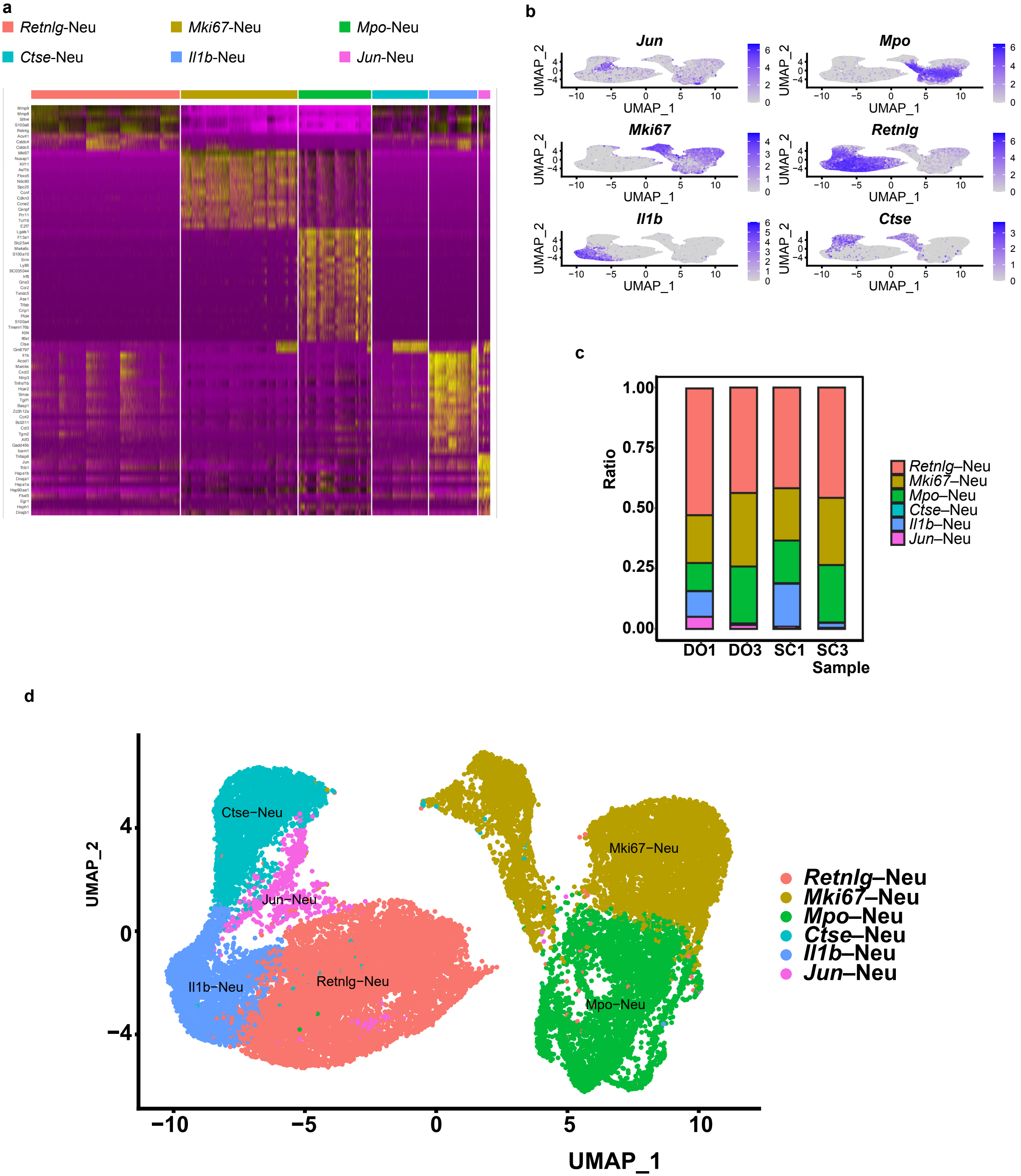

a
Retnlg-Neu
Mki67-Neu
Mpo-Neu
Ctse-Neu
Il1b-Neu
Jun-Neu
b
Jun
Mpo
Mki67
Retnlg
Il1b
Ctse
UMAP_1
UMAP_2
c
Ratio
1.00
0.75
0.50
0.25
0.00
DO1
DO3
SC1
SC3
Sample
Retnlg–Neu
Mki67–Neu
Mpo–Neu
Ctse–Neu
Il1b–Neu
Jun–Neu
d
Ctse−Neu
Jun−Neu
Il1b−Neu
Retnlg−Neu
Mki67−Neu
Mpo−Neu
UMAP_1
UMAP_2
Retnlg–Neu
Mki67–Neu
Mpo–Neu
Ctse–Neu
Il1b–Neu
Jun–Neu
