# Supplementary material for: CREB3L1-Associated Stromal Expression During Mechanical Remodeling of the Mouse Zygomaticomaxillary Suture
Source: Biology (Basel). 2026 Oct 23;15(21):1918. doi: 10.3390/biology15211918 (PMC13762063; doi:10.3390/biology15211918)
Supplement: Supplementary file 1 [file biology-15-01918-s001.zip › Supplementary_Figures.zip!/Supplementary_Figures/Figure_S7.pdf]

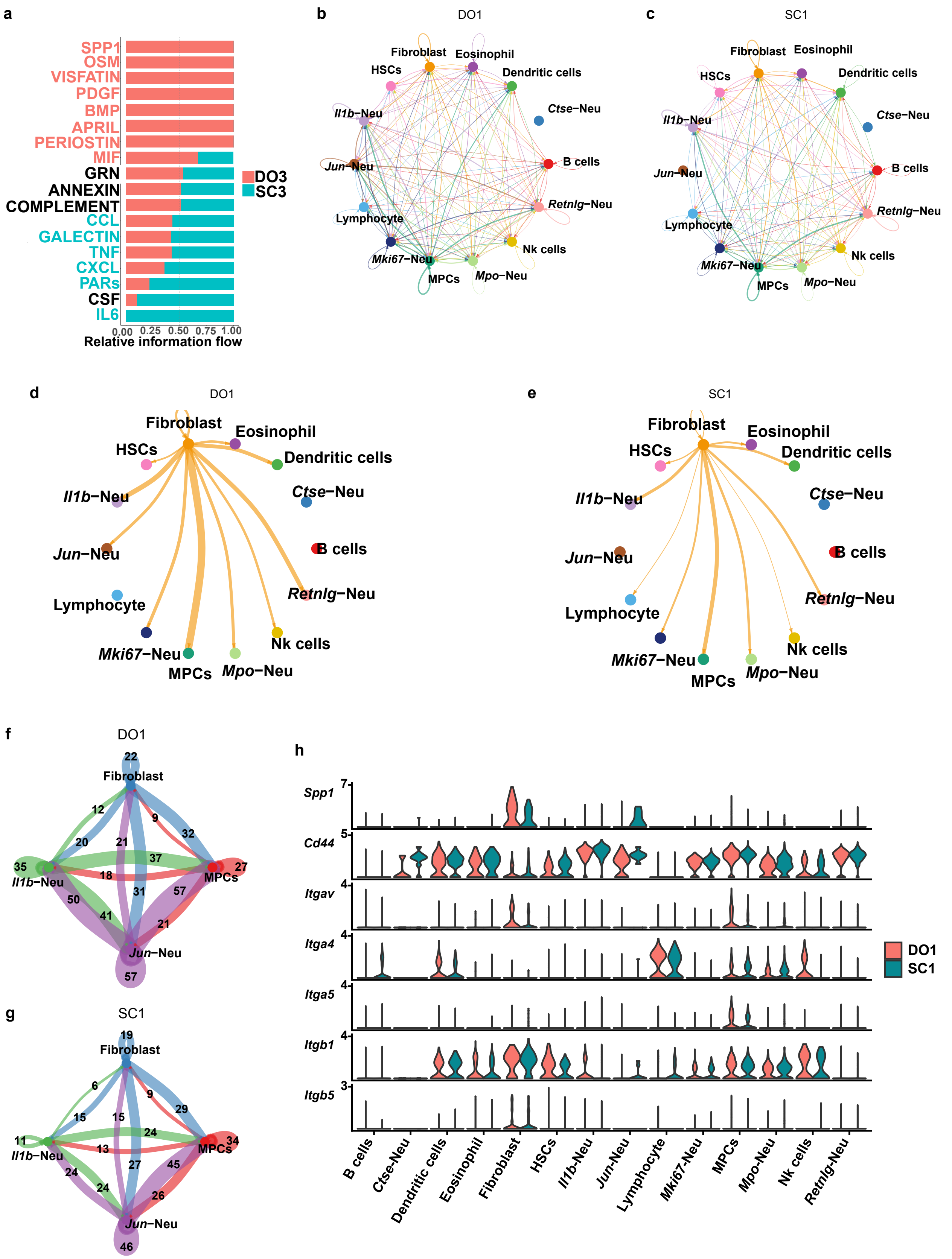
a
SPP1
OSM
VISFATIN
PDGF
BMP
APRIL
PERIOSTIN
MIF
GRN
ANNEXIN
COMPLEMENT
CCL
GALECTIN
TNF
CXCL
PARs
CSF
IL6
DO3
SC3
0.00 0.25 0.50 0.75 1.00
Relative information flow
b
DO1
c
SC1
Fibroblast
Eosinophil
HSCs
Dendritic cells
Il1b−Neu
Ctse−Neu
Jun−Neu
B cells
Retnlg−Neu
Lymphocyte
Nk cells
Mki67−Neu
MPCs
Mpo−Neu
d
DO1
e
SC1
f
DO1
g
SC1
h
Spp1
Cd44
Itgav
Itga4
Itga5
Itgb1
Itgb5
DO1
SC1
B cells
Ctse-Neu
Dendritic cells
Eosinophil
Fibroblast
HSCs
Il1b-Neu
Jun-Neu
Lymphocyte
Mki67-Neu
MPCs
Mpo-Neu
Nk cells
Retnlg-Neu
